# Screening for *Fusarium* Antagonistic Bacteria From Contrasting Niches Designated the Endophyte *Bacillus halotolerans* as Plant Warden Against *Fusarium*

**DOI:** 10.3389/fmicb.2018.03236

**Published:** 2019-01-11

**Authors:** Houda Ben Slama, Hafsa Cherif-Silini, Ali Chenari Bouket, Mallique Qader, Allaoua Silini, Bilal Yahiaoui, Faizah N. Alenezi, Lenka Luptakova, Mohamed Ali Triki, Armelle Vallat, Tomasz Oszako, Mostafa E. Rateb, Lassaad Belbahri

**Affiliations:** ^1^NextBiotech, Agareb, Tunisia; ^2^Institut de l’Olivier Sfax, Sfax, Tunisia; ^3^Laboratory of Applied Microbiology, Department of Microbiology, Faculty of Natural and Life Sciences, University Ferhat Abbas of Setif, Setif, Algeria; ^4^School of Science and Sport, University of the West of Scotland, Paisley, United Kingdom; ^5^National Institute of Fundamental Studies, Kandy, Sri Lanka; ^6^Department of Biology and Genetics, Institute of Biology, Zoology and Radiobiology, University of Veterinary Medicine and Pharmacy in Košice, Košice, Slovakia; ^7^Neuchatel Platform of Analytical Chemistry, Institute of Chemistry, University of Neuchâtel, Neuchâtel, Switzerland; ^8^Department of Forest Protection of the Forest Research Institute in Sękocin Stary, Raszyn, Poland; ^9^Laboratory of Soil Biology, University of Neuchâtel, Neuchâtel, Switzerland

**Keywords:** *Phoenix dactylifera*, *Fusarium oxysporum* f. sp. *albedinis*, Bayoud disease, endophyte, secretome, volatilome, comparative genomics, pan genome

## Abstract

Date palm (*Phoenix dactylifera* L.) plantations in North Africa are nowadays threatened with the spread of the Bayoud disease caused by *Fusarium oxysporum* f. sp. *albedinis*, already responsible for destroying date production in other infected areas, mainly in Morocco. Biological control holds great promise for sustainable and environmental-friendly management of the disease. In this study, the additional benefits to agricultural ecosystems of using plant growth promoting rhizobacteria (PGPR) or endophytes are addressed. First, PGPR or endophytes can offer an interesting bio-fertilization, meaning that it can add another layer to the sustainability of the approach. Additionally, screening of contrasting niches can yield bacterial actors that could represent wardens against whole genera or groups of plant pathogenic agents thriving in semi-arid to arid ecosystems. Using this strategy, we recovered four bacterial isolates, designated BFOA1, BFOA2, BFOA3 and BFOA4, that proved very active against *F. oxysporum* f. sp. *albedinis*. BFOA1–BFOA4 proved also active against 16 *Fusarium* isolates belonging to four species: *F. oxysporum* (with strains phytopathogenic of *Olea europaea* and tomato), *F. solani* (with different strains attacking *O. europaea* and potato), *F. acuminatum* (pathogenic on *O. europaea*) and *F. chlamydosporum* (phytopathogenic of *O. europaea*). BFOA1–BFOA4 bacterial isolates exhibited strong activities against another four major phytopathogens: *Botrytis cinerea*, *Alternaria alternata*, *Phytophthora infestans*, and *Rhizoctonia bataticola.* Isolates BFOA1–BFOA4 had the ability to grow at temperatures up to 35°C, pH range of 5–10, and tolerate high concentrations of NaCl and up to 30% PEG. The isolates also showed relevant direct and indirect PGP features, including growth on nitrogen-free medium, phosphate solubilization and auxin biosynthesis, as well as resistance to metal and xenobiotic stress. Phylogenomic analysis of BFOA1–BFOA4 isolates indicated that they all belong to *Bacillus halotolerans*, which could therefore considered as a warden against *Fusarium* infection in plants. Comparative genomics allowed us to functionally describe the open pan genome of *B. halotolerans* and LC-HRMS and GCMS analyses, enabling the description of diverse secondary metabolites including pulegone, 2-undecanone, and germacrene D, with important antimicrobial and insecticidal properties. In conclusion, *B. halotolerans* could be used as an efficient bio-fertilizer and bio-control agent in semi-arid and arid ecosystems.

## Introduction

Biological control relies on screening for pest or pathogen antagonists or natural enemies, originating from its area of spread or areas that closely match the climate and soil type of the infested region by the pest or pathogen. This allows researchers to match the definable environmental limits for the existence of the biological control agent (Debbi et al., 2018; Gomez-Lama Cabanas et al., 2018; Guardado-Valdivia et al., 2018). Despite the selection of definable environmental limits for the existence of the biological control agent, some biocontrol agents fail to compete or colonize the rhizosphere of inoculated plants (Mefteh et al., 2017).

Therefore, the new approach is to make use of PGPR and endophytes, having the potential of a high rhizosphere competence, as biological control agents and therefore allowing successful biocontrol strategies (Santoyo et al., 2016; Mefteh et al., 2017; El-Sayed et al., 2018; Kosawang et al., 2018; Schreiter et al., 2018). This strategy proved successful in extreme habitats that expose plants to high levels of abiotic stresses, where few species are able to thrive. Therefore, the ecological concept of habitat-adapted symbiosis has been proposed (Rodriguez et al., 2008; Soares et al., 2016). This concept is currently undergoing expansion and holds promises to mitigate plant stress in extreme habitats in addition to the classical role of pest or pathogen management (Rekik et al., 2017; Cherif-Silini et al., 2012).

Date palm (*Phoenix dactylifera* L.), a widely distributed tree throughout the Middle East and North Africa, is cultivated for its edible sweet fruit. The tree represents a source of raw materials for construction, consumption and other life functions. Different parts of date palm such as the fruit, pollen and date palm sap “Lagmi” are also popular in folk medicine (Abdennabi et al., 2016; Daoud et al., 2017). In the Middle East and North Africa, date palm trees are more than a source of income, they represent a cultural emblem of the region and a cornerstone in the fragile oasis ecosystems through allowing oasis cultivation systems. Loss of date palm trees is supposed to dramatically impact arid regions inhabiting populations and may trigger massive economic migration to urban cities (Mefteh et al., 2017). Unfortunately, date palm cultivation is under an unprecedented threat due to infection with *Fusarium oxysporum* f. sp. *albedinis*, considered as one of the most serious pathogens of date palm ever recorded (Jeger et al., 2018). *F. oxysporum* f. sp. *albedinis* is currently thriving in the oases of Morocco, Algeria, and Mauritania on its major host, *P. dactylifera*, the only *Phoenix* species known to be affected by *F. oxysporum* f. sp. *albedinis*. This pathogen could potentially enter the EU through increasing international traffic and trade of plants or plant products (Jeger et al., 2018). The pathogen meets all the criteria for consideration as a potential EU quarantine pest, given its putative high environmental consequences in the palm grove of Elche (Spain), declared as World Heritage Site by UNESCO in 2000. Current management procedures, while correctly applied mainly in Morocco and Algeria, failed to eradicate the pathogen. Therefore, approaches targeting the pathogen through strict application of quarantine measures do not seem to enable eradication as promised by the early studies. Biological control is a promising approach that proved effective in the control of many plant pathogen and plant pest ecosystems (El Hassni et al., 2007; Dihazi et al., 2012; Abad et al., 2014; Alenezi et al., 2017; Mefteh et al., 2017, 2018; Cherrad et al., 2018).

Apart from the role of pest or pathogen management, traditionally attributed to biocontrol agents (Gao et al., 2018), second generation biocontrol agents based on the use of PGPR or endophytes provide new biocontrol agents with multifaceted roles. They are endowed with numerous other functions such as plant growth promotion, plant yield enhancement, soil bio-fertilization, abiotic stress and drought mitigation (Belbahri et al., 2017; Bamisile et al., 2018; Orozco-Mosqueda et al., 2018; Prabhukarthikeyan et al., 2018). This is particularly relevant for biocontrol agents suitable for dry climates based on the meta-analysis conducted by Schutz et al. (2018) to quantify benefits of bio-fertilizers in terms of yield increase, nitrogen and phosphorus use efficiency. These data were based on 171 peer-reviewed publications, which indicated the superiority of bio-fertilizer performance in dry climates over other climatic regions (yield response: dry climate +20.0 ± 1.7%).

In addition to these conceptual changes that are driving the field of biocontrol, recent technological breakthroughs are providing new tools that are actively applied to study PGPR and endophytes (Olson et al., 2012; Luchi et al., 2013; Prospero et al., 2013; Alenezi et al., 2015a,b, 2016a,b; Belbahri et al., 2015, 2017; Sellami et al., 2016). PGPR or endophytes are more deeply studied using genomics or metagenomics, and their secretomes and volatilomes are widely characterized using GCMS and LCMS technologies, which are providing new insights into the PGPR and endophyte biocontrol agent arsenal for counteracting pathogens (Bailly and Weisskopf, 2017). Additionally, software that provide mining of secondary metabolite gene clusters are available to provide the link between secondary products identified in the secreted fractions of the bacteria and their secondary metabolites’ genomic potential (Skinnider et al., 2015; Weber et al., 2015). These tools are helpful in characterizing the unexploited, eco-friendly chemical resources of these current-generation biocontrol agents.

In some cases, biocontrol agents would be either active in a given habitat or climate or on diverse pathogens thriving on different hosts (Lara and Belbahri, 2011; Mefteh et al., 2017). In this report, we aimed to use PGPR and endophytes from contrasting niches, in terms of geographic location and colonizing plants, to recover biocontrol agents against *F. oxysporum* f. sp. *albedinis* that are rhizosphere competent and possess a wide range of biocontrol abilities against diseases in multiple hosts thriving in these habitats. We managed to isolate biocontrol agents from the two niches that are active against *F. oxysporum* f. sp. *albedinis*, as well as against other *Fusarium* spp. thriving on multiple hosts (date palm, olive trees, tomato, and potato). Their genomic identification indicated that they belong to the same species, *B. halotolerans*. We have qualified this species as a plant warden against the *Fusarium* genus. Our four isolates have been targeted by genome sequencing and LC-HRMS and GCMS analyses of their secretome and volatilome. They also served to set a comparative genomic approach of *B. halotolerans*.

## Materials and Methods

### Sampling Locations

Two locations in Tunisia and Algeria within the semi-arid climate were targeted during sampling processes. The Tunisian sampling site, representing a coastal saline depression (34° 25′ 59.99″ N, 10° 10′ 60.00″ E), faces the Mediterranean Sea. It is characterized by a mild climate with relatively high humidity. Six plants of *Limoniastrum monopetalum* (L.) Boiss. were collected and roots without visible damage were transported safely to the laboratory and stored at 4°C until their use for endophyte isolation. The Algerian sampling site was located in the North of Setif and represents a semi-arid soil of the Ouricia region [36°17′40.5″N 25′32.8″E, pH 7.90, electrical conductivity (EC) = 1.3 ms/cm]. The rhizosphere of wheat plants growing in the sampling site was collected with an intact root system. The samples were placed in plastic bags and stored at 4°C during the transportation to the Laboratory of Applied Microbiology (University Ferhat Abbas, Setif, Algeria) where they were stored under the same conditions for further analysis.

### Isolation of Endophytic Bacteria From *L. monopetalum* Collected in Tunisia

Roots (primary and secondary) were thoroughly washed using tap water and subjected to surface disinfestation. Briefly, roots were placed in 70% ethanol for 3 min, 1% sodium hypochlorite for 1 min and then washed three times in sterile distilled water for 3 min each time. Absence of microbial growth from the water-wash on tryptic soy agar (TSA) Petri plates was considered as proof of success for surface sterilization. Sterilized roots were then sliced to tiny pieces (5 mm^2^ approximately) and placed on the surface of TSA Petri dishes. After 2–3 days incubation at 30°C emerging colonies were transferred to fresh Petri dishes to establish pure cultures. More than 120 colonies have been collected and used in subsequent screenings.

### Isolation of Bacteria From the Rhizosphere of Durum Wheat Collected in Algeria

Approximately 1 g of strongly adhering soil to roots of wheat plants was incubated in 10 mL of sterile distilled water in an Erlenmeyer and vigorously shacked at 200 rpm for 30 min. Aliquots of resulting diluted rhizospheric soil extract were plated on TSA medium Petri plates. Plates were then incubated at 30°C for 2 days and the growing colonies were transferred to new Petri dishes in order to establish bacterial pure cultures. More than 40 colonies were collected and used in subsequent screenings.

### Isolation of BFOA1–BFOA4 Isolates

The two bacterial collections isolated from the roots of *L. monopetalum* (L.) Boiss collected from the Tunisian site and from the wheat rhizosphere growing in Setif (Algeria) were tested for antagonism toward *F. oxysporum* f. sp. *albedinis* as described in the section “Antifungal assays.” Four bacterial isolates showing high *F. oxysporum* f. sp. *albedinis* inhibition were named BFOA1–BFOA4 and used in the time course of this study.

### Bacterial Isolates and Fungal Strains and Growth Conditions

All bacterial and fungal isolates used in the current study have been described in the Supplementary Tables S1, S2. Unless indicated, all bacterial and fungal cultures were cultured on TSA and potato dextrose agar (PDA) media, respectively.

### Antifungal Activity Assays

The ability of isolates BFOA1–BFOA4 to inhibit the growth of phytopathogenic fungi listed in Supplementary Table S2 was studied. All isolates were obtained from the Laboratory of Applied Microbiology (Faculty of Nature and Life Sciences, University Ferhat Abbas of Setif) culture collection. Antagonism of bacterial isolates toward phytopathogenic fungi was performed *in vitro* using PDA plates. Agar disks of the studied fungal culture were applied on the Petri dish 3 cm apart from each bacterial culture spot. A negative control consisting of fungal agar disks in the absence of bacterial culture spots was also conducted. The Petri dishes were then incubated at 30°C for 7 days. The percentage of inhibition of fungal growth was calculated by the following formula proposed by Alenezi et al. (2016b).

1−(a/b)×100%

Where *a* is the distance between fungal growth edge (from the bacterial side) and the bacterial isolate growth edge (from the fungus side) and *b* is the distance between the fungal upper growth edge and the upper edge of the control Petri dish.

### Effects of *Bacillus halotolerans* BFOA4 Treatment on *F. oxysporum* f. sp. *radicis-lycopersici* Strain FORL Disease Severity on Tomato Fruits

*In vivo* antifungal activity of *Bacillus halotolerans* (BFOA4) against *F. oxysporum* f. sp. *radicis-lycopersici* strain FORL infection was studied on healthy tomato fruits. They were surface sterilized by immersion into 3% sodium hypochlorite (NaOCl) for 15 min, rinsed several times with sterile distilled water and dried under filter-sterilized air flow. In each tomato fruit one artificial well was created with sterile punch 4 mm wide and deep. Then, treatments were done according to the protocol described by Yangui et al. (2013).

In preventative or curative treatment, an agar disk containing *B. halotolerans* was added 24 h before or after infection with the FORL fungus. The concomitant treatment received, in addition to the agar disk of *B. halotolerans*, an agar disk containing mycelial fragments of FORL simultaneously. Additionally, three different controls were done: one control treated with bacteria, the other control infected with fungus and the last one served to measure the effect of the incision. Tomato fruits were stored individually in plastic bags and incubated for 7 days at 25°C. Relative humidity of about 85% was ensured by the introduction of cotton containing sterile distilled water in each bag. The experiment was repeated four times. The penetration diameter was calculated after the incubation period following the formula of Lapwood et al. (1984):

P(mm)=[W/2+(D−P)]/2

Where W is the width of the rot (mm), D is the depth of the rot (mm) and P is the depth of the inoculation well (mm).

Therefore, the percentage of inhibition of the rot extension was calculated according to the present formula:

Inhibition of the extension(%)=[(Ti−Tr)/Ti]×100

Where Ti (mm) is the positive control (inoculated and non-treated fruit) and Tr (mm) is the inoculated and treated fruit.

### Effect of Salt, Temperature, PEG and pH on BFOA1–BFOA4 Bacterial Isolates

The ability of BFOA1–BFOA4 isolates to grow at different concentrations of salt, PEG, and at different pH values was performed on LB medium supplemented with NaCl (0, 200, 400, 600, 800, 1000, and 1200 mM), PEG (10, 20, and 30%) and at different pH (4, 7, 9, and 11) according to Cherif-Silini et al. (2012). The media were inoculated with 100 μL of bacterial cultures and incubated at 30°C for 2 days. Bacterial growth was then studied by measuring the optical density at 600 nm using a spectrophotometer (Spectronic Genesys 20 Visible Spectrophotometer, Setif, Algeria). The ability of BFOA1–BFOA4 isolates to tolerate different temperatures was verified by incubating LB broth media inoculated with 100 μL of bacterial cultures at 4, 30, 37, 45, and 55° C for 2 days and was determined by optical density measurement at 600 nm using a spectrophotometer (Spectronic Genesys 20, Setif, Algeria). Three technical and biological repetitions were performed. All spectrophotometric measurements were performed in triplicate.

## Measurement of PGP Activities of BFOA1–BFOA4 Bacterial Isolates

### Direct PGPR Activities

At least three biological and technical repetitions have been performed for all direct PGP activities targeted in this study. All used protocols were described in detail in Cherif-Silini et al. (2012).

### Growth on Nitrogen Free Medium

Following the protocol described by Cherif-Silini et al. (2012), growth of isolates BFOA1–BFOA4 on nitrogen free medium was checked by its ability to grow on the Winogradsky Salt (WS) medium.

### Phosphate Solubilization

The procedure of Gaur (1990) was used in this study to assay phosphate the solubilization ability of isolates BFOA1–BFOA4. Approximately 10 μL of BFOA1–BFOA4 bacterial cultures were spotted on the surface of the Pikovskaya (PVK) medium containing tricalcium phosphate as the sole source of phosphate. After 7 days of incubation at 30°C, the appearance of a halo around the colony indicated phosphate solubilization. Phosphate solubilization ability was directly reflected by halo diameter size. For quantitative analysis of phosphate solubilization, a tricalcium phosphate solubilization assay in liquid medium was conducted by inoculating 100 μL of BFOA1–BFOA4 bacterial cultures in PVK medium and incubating the resulting cultures at 30°C for 4 days. After centrifugation for 15 min at 3000 rpm, soluble phosphate was measured in the supernatant according to Olsen and Sommers (1982). As suggested in this procedure, absorbance measured at 610 nm was compared to a standard calibration curve using KH_2_PO_4_ solution.

### Siderophores Production

The procedure of Schwyn and Neilands (1987) using Chrome Azurol S (CAS) media was implied in this study to perform semi-quantitative production of siderophore. Briefly, 10 μL bacterial cultures of BFOA1–BFOA4 isolates were inoculated on iron-free King B solid media and incubated at 30°C for 24 h. 15 mL of CAS agar were then poured on the BFOA1–BFOA4 bacterial cultures and the plates checked after a few hours for the appearance of a halo around bacterial colonies with a color change from blue to orange. Halo diameter was then calculated by subtracting the diameter of the colony from the total diameter of the halo and the colony. Siderophore production was also assessed quantitatively by inoculating King B media with 100 μL of *B. halotolerans* BFOA1–BFOA4 bacterial cultures and incubation at 30°C for 2 days. After centrifugation at 3000 rpm for 30 min, the supernatants (500 μL) were then mixed with 500 μL of CAS solution and incubated for 20 min. Optical density (OD) was measured at 630 nm and siderophore production (SP) was evaluated using the Gokarn (2010) formula and expressed as a percentage:

SP(%)=OD of the sample/OD of the control (CAS solution)

### Indole Acetic Acid (IAA) Production

Production of IAA was assayed using the procedure of Loper and Schroth (1986). Briefly, Winogradsky broth supplemented with 2 g/L of tryptophan was inoculated with 100 μL of BFOA1–BFOA4 bacterial cultures and incubated at 30°C for 2 days. The resulting bacterial cultures were then centrifuged at 3000 rpm for 20 min. The resulting supernatants (2 mL) were then mixed with 4 mL of Salkowski reagent (50 mL of 35% perchloric acid and 1 mL of 0.5 M FeCl_3_) and OD measured at 530 nm. IAA concentration was determined by comparing OD to the standard calibration curve obtained using IAA solution ranging from 0 to 10^-3^ M.

### Indirect PGP Activities

Indirect PGP activities presented in this study were the average of triplicate assays. All used protocols were described in detail in Cherif-Silini et al. (2012).

### Protease Production

BFOA1–BFOA4 isolates were inoculated on skimmed milk agar media (Loper and Schroth, 1986). Plates were then incubated at 30°C for 2–3 days and protease activity recorded by the development of a clear zone (halo) around the colonies, indicating proteins were hydrolyzed by the bacteria.

### Chitinase Production

Growth on chitin agar plates following the procedure described by Cappuccino and Sherman (1992) was used to evaluate the chitinase activity of the BFOA1–BFOA4 isolates. After incubating the plates at 30°C for 5 days, chitinase activity was revealed by the development of a clear zone (halo) around the colonies.

### Cellulase Production

Cellulase production ability was assessed by inoculation of isolates BFOA1–BFOA4 on nutrient agar media supplemented with 10 g/L carboxymethyl cellulose. Inoculation was performed by spotting 5 μL of bacterial cultures on the medium and subsequent Petri dish incubation at 30°C for 2 days. After pouring 0.1% Red Congo solution on the surface of the Petri dishes, destaining using 1 M NaCl solution was performed. Development of a clear halo around the colonies indicated a positive reaction and the production of cellulase by the bacteria.

### Amylase Production

Amylase production ability was assessed by inoculation of isolates BFOA1–BFOA4 on nutrient agar media supplemented with 0.5% soluble starch. Inoculation was performed by spotting 5 μL of bacterial culture on the medium and subsequent Petri dish incubation at 30°C for 2 days. After pouring an iodine solution (0.3 g iodine and 0.6 g KI/L) on the surface of the Petri plates, clear halo development around the colonies indicated a positive reaction and the production of amylase and degradation of starch by the bacteria.

### ACC Deaminase Production

The ACC deaminase production of bacterial cultures of BFOA1–BFOA4 was confirmed in accordance with Glick et al. (1994) by their growth on minimal media containing ACC as the sole nitrogen source. After culture incubation at 30°C for 2 days, OD was measured at 540 nm by spectrophotometer (Spectronic Genesys 20, Setif, Algeria) and ACC deaminase activity was considered positive when higher than the OD of a mineral solution (MgSO_4_).

### NH_3_ Production

Ammoniac production was estimated essentially as described by Cappuccino and Sherman (1992). Bacterial cultures of isolates BFOA1–BFOA4 (100 μL) were inoculated on Peptone water and cultures incubated at 30°C for 2 days. After the addition of Nessler’s reagent (0.5 mL), the appearance of a yellowish to brownish color indicated NH_3_ production.

### Hydrogen Cyanide (HCN) Production

HCN production by isolates BFOA1–BFOA4 was monitored following the procedure described by Lorck (1948). Briefly, bacterial cultures of isolates BFOA1–BFOA4 were inoculated on HCN medium (Nutrient agar supplemented with 4.4 g/L of glycine). Cyanide production was detected using picrate/Na_2_CO_3_ impregnated Whatman paper (9 mm in diam.) fixed to the underside of the plate lid. Plates were sealed with Parafilm and subsequently incubated at 30°C for 4 days. Orange to red color development on the Whatman paper indicated the production of HCN.

### Resistance of Bacterial Isolates BFOA1–BFOA4 to Metal Stress

Resistance to lead, cadmium, cobalt and mercury of the isolates BFOA1–BFOA4 was assessed by spotting 10 μL of bacterial culture suspension (10^8^ cells/mL) on LB solid media supplemented with the different metal salts. PbCl_2_, Cd(NO_3_)_2_, CoCl2 and HgCl_2_ were used at 50, 100, 250, 500, and 1000 ppm for examining the resistance of the different bacterial isolates to metal stress. After incubation of the cultures at 30°C for 72 h, the minimum inhibitory concentration (MIC) was set as the smallest concentration of the metal considered that inhibits bacterial growth (Xiumei et al., 2014).

### Antibiotic Resistance of Bacterial Isolates BFOA1–BFOA4

Resistance/sensitivity of isolates BFOA1–BFOA4 to antibiotics was performed using the agar-diffusion technique. Bacterial inocula adjusted to 10^7^ cells/mL were spread as evenly as possible with a sterile swab on the surface of the Mueller-Hinton agar plates. Antimicrobial susceptibility test disks were then placed on the Petri dishes’ surfaces. Antibiotics used in the present study were amoxicillin (AMX, 30 μg), amoxicillin/clavulanic acid (AMC, 30 μg), aztreonam (ATM, 30 μg), cephalexin (CXN, 30 μg), cefepime (FEP, 30 μg), cefotaxime (CTX, 30 μg), ceftazidim (CAZ, 30 μg), ceftriaxon (CRO, 30 μg), imipenem (IPM, 10 μg), methicillin [ME, 5 International Unit (IU)], amikacin (AKN, 30 μg), gentamycin (GEN, 10 IU), streptomycin (SMN, 10 IU), doxycycline (DO, 30 μg), minocycline (MIN, 30 μg), tetracycline (TET, 30 IU), nalidixic acid (NAL, 30 μg), ciprofloxacin (CIP, 5 μg), chloramphenicol (C, 30 μg), erythromycin (ERY, 15 IU), fosfomycin (FSF, 50 μg), trimethoprim/sulfamethoxazole (SXT, 25 μg), and vancomycin (VAN, 30 μg). Petri dishes were then incubated at 30°C for 24 h and the inhibition diameters calculated and compared to standards in order to evaluate the susceptibility/resistance of the given bacterial isolates.

### Bacterial DNA Extraction and Amplification

The UltraClean^®^ Microbial DNA Isolation Kit (QIAGEN, Basel, Switzerland) was used to extract bacterial isolates’ high molecular weight genomic DNA suitable for whole genome sequencing according to manufacturer recommendations. Qubit Fluorometric Quantitation (Thermo Fisher, Switzerland) was used to quantify genomic DNA, while DNA integrity and quality were assessed by visual inspection through 1.5% agarose gel electrophoresis. Molecular identification of the isolates was performed through amplification of the 16S rRNA region using primers fD1 (5′ AGAGTTTGATCCTGGCTCAG 3′) and rP2 (5′ ACGGCTACCTTGTTACGACTT 3′) (Weisburg et al., 1991; Mlaik et al., 2015). PCR amplifications were carried out in a total volume of 50 μL, containing 5 μL 10× Ex Taq buffer (20 mM Tris–HCl, pH 8.0, 100 mM KCl), 4 μL 2.5 mM dNTP mixture, 0.5 μM of each primer, 1.25 units Taq DNA polymerase (Takara Bio, Ohtsu, Japan) and 10 ng genomic DNA. The following cycling conditions were used: initial denaturation step at 95°C for 1 min followed by 35 cycles (denaturation at 94°C for 30 s, annealing at 55°C for 30 s and extension at 72°C for 20 s and final extension step at 72°C for 5 min) using a Biometra Tone thermal cycler (Labgene, Chatel-Saint-Denis, Switzerland). A minelute PCR Purification Kit (Qiagen, Basel, Switzerland) was then used to purify the resulting PCR amplicons according to the manufacturer’s specifications.

### DNA Sequencing and Phylogenetic Analysis

16S rRNA purified PCR amplicons were sequenced in both directions using a BigDye^®^ Terminator v. 3.1 cycle sequencing kit and primers fD1 and rP2. ABI 3130 XL, available at the iGE3 [Institute of Genetics and Genomics in Geneva, University of Geneva Medical Center (CMU), Switzerland], was used to resolve sequencing reactions. Manual editing of raw sequence files was then performed using SeqMan^TM^II (DNASTAR, Madison, WI, United States) to generate consensus sequences. The consensus sequence was then blasted against the NCBI’s GenBank sequence database to identify their closest species relatives. Exact phylogenetic position of the different isolates BFOA1–BFOA4 was additionally ascertained by phylogenetic analysis of the isolates’ sequences with their closest relatives retrieved from GenBank. Briefly, collected sequences were aligned using the multiple sequence alignment web-based program MAFFT (Katoh and Toh, 2008) and used to generate phylogenetic trees based on the Maximum Likelihood (ML) algorithm (Felsenstein, 1981). MEGA v.6 (Tamura et al., 2013) with computed evolutionary distances using the Kimura 2-parameter (Kimura, 1980). The validity of branches in the resulting trees was evaluated by bootstrap resampling support of the data sets with 1000 replications.

### Bacterial Genome Sequencing Assembly and Annotation

The four genomes of bacterial isolates BFOA1, BFOA2, BFOA3, and BFOA4 (Supplementary Table S1) were sequenced using the facilities available at the iGE3 genomics platform of the University of Geneva^1^. Briefly, genomic DNA was used to generate a sequencing library using Illumina’s TruSeq sample preparation reagents. The BFOA1–BFOA4 MiSeq sequencing starting materials were then inserted into the reagents cartridge and loaded on the instrument along with the flow cell. Assembly was then attempted on resulting reads after low quality reads filtering. Bacterial genome sequences (BFOA1, BFOA2, BFOA3, and BFOA4) were deposited under accession numbers PVXA00000000, PVXB00000000, PVXC00000000, and PVXD00000000, respectively.

### Selection and Phylogenomic Analysis of *B. halotolerans* Isolates

All publicly available genomes of *B. halotolerans* isolates, having more than 98% 16S rRNA gene sequence similarity with the type strain DSM 8802, were retrieved from GenBank (Supplementary Table S1). The CheckM program (v1.0.9 released on December 11, 2017; Parks et al., 2015) allowed assessment of completeness and contamination rates of the collected *B. halotolerans* genomes. All genomes showed high quality draft genome sequences with ≥90.0% completeness and ≤10.0% contamination, justifying their use in the different analyses performed in the study. Genome-based Average Nucleotide Identity (ANI) and Genome to Genome Distance (GGD) values were used to select genomes phylogenomically belonging to the species *B. halotolerans*. They were estimated using the ANI online server^2^ and the server-based genome-to-genome distance calculator (V. 2.1^3^) according to Yoon et al. (2017) and Meier-Kolthoff et al. (2013), respectively. For ANI and GGD analysis, species and sub-species cut-off were those suggested by default analysis (95–96% and 70%, respectively). Whole genome alignments were conducted using the reference sequence alignment-based phylogeny builder (REALPHY)^4^ (Bertels et al., 2014). A neighbor-joining (NJ) algorithm (Saitou and Nei, 1987), as implemented in MEGA v.6 (Tamura et al., 2013) with evolutionary distances computed using the Kimura 2-parameter model (Kimura, 1980), was used to build the phylogenomic tree. The validity of branches in the resulting tree was evaluated by bootstrap re-sampling support of the data sets with 1000 replications.

### Homology-Based Mining of Genes Contributing to Plant-Beneficial Functions in *B. halotolerans*

Genes encoding for nutrient acquisition, root colonization and growth promotion factors, plant growth-promoting traits (hormones), plant protection from oxidative stress (antioxidant enzymes), plant induction of disease resistance, antibiotics and related compounds, resistance to drugs, resistance to heavy metals and degradation of aromatic compounds were mined in the genomes of the *B. halotolerans* isolates collection as described by Belbahri et al. (2017).

### Comparative Genomics Analysis of *Bacillus halotolerans* Isolates

Pan- and core-genomes of the *B. halotolerans* isolates collection (Supplementary Table S1) were computed using the Chaudhari et al. (2016) BPGA pipeline, with a 50% sequence identity cut-off. Assignment of core and pan-genomes’ functional genes into COG categories within the BPGA pipeline was performed using the USEARCH program against the standard COG database. For functional annotation based on KEGG Orthology (KO), predicted proteins derived from the *B. halotolerans* genomes were subjected to BlastKOALA analysis^5^ (Kanehisa et al., 2016).

### Secondary Metabolite Clusters Identification Using antiSMASH, PRISM, NapDos, NP.search, and Bagel3

The annotated draft genome sequence files of the *B. halotolerans* isolate collection (Supplementary Table S1) were subjected to secondary metabolite gene cluster analysis using antiSMASH 3.0 (Weber et al., 2015), prediction informatics for secondary metabolomes (PRISM) (Skinnider et al., 2015), NapDos (Ziemert et al., 2012), NP.search (Li et al., 2009), and the bacteriocin-specific software BAGEL3 (Van Heel et al., 2013).

### Identification of Core Genome and Accessory Genomes of the Isolate Collection

*Bacillus halotolerans* core genome, defined as those sequences present in nearly all genomes from the isolates collection (Supplementary Table S1), and accessory genomes, defined as those sequences present only in some isolates of the collection, were determined using Spine and Agent, respectively (Ozer et al., 2014).

### Preparation of Bacterial Isolates BFOA1–BFOA4 Extracts for LC-HRMS Analysis

After small scale fermentation of each bacterium, about 50 g/L diaion HP20 resin was added to the fermentation flask, left shaking for 6 h, then centrifuged at 10000 rpm for 5 min. The precipitate was then extracted with methanol twice and the combined methanolic extract was evaporated under vacuum to a residue. Residue (1 mg) was accurately weighed and dissolved in 10 mL methanol and about 1 mL of this solution was filtered through a 0.2 μm PTFE filter into an HPLC vial where it was submitted to L-HRCMS analysis.

### LCMS Analysis of Extracts From Bacterial Isolates BFOA1–BFOA4

The UHPLC-HRMS experiments were performed on a Synapt G2 high resolution mass spectrometer coupled to an Acquity UPLCTM (Waters, Milford, MA, United States). Separation of the compounds was achieved on an Acquity BEH C18 column with 50 mm × 2.1 mm i.d., 1.7 μm particle size with a guard column of identical phase chemistry (Waters, Milford, MA, United Sttaes). The mobile phase was 0.05% formic acid in water (A)/acetonitrile (B) and the following gradient elution program was used: 0 min 5% B; 5–70% B in 6 min; 70–100% B in 2 min, holding at 100% during 2 min, and re-equilibration at 5% B for 1.1 min. The flow rate was set to 400 μL/min, the injection volume was 2.5 μL, and the column temperature was maintained at 25°C. For MS detection, ionization was performed in positive and negative ESI modes using a mass scan range from 85 to 2000 Da. Experimental source parameters were performed as follows: capillary voltage 2.8 kV in positive mode and 2 kV in negative mode, sampling cone 25 V, source and desolvation temperatures 120 and 500°C, respectively, and desolvation gas flow 800 L/Hr. Data was processed using MestreNova 11.0 suite (Mestrelab, Santiago de Compostela, Spain).

### Preparation of Extracts From Bacterial Isolates BFOA1–BFOA4 for GCMS Analysis

After small scale fermentation of each bacterium, about 50 g/L diaion HP20 resin was added to the fermentation flask, left shaking for 6 h, then centrifuged at 10000 rpm for 5 min. The precipitate was then extracted with methanol twice and the combined methanolic extract was evaporated under vacuum to a residue. The residue was re-dissolved in 10 mL methanol fractioned with 10 mL n-hexane in a separating funnel twice. The hexane extract was evaporated and 1 mg of the residue was dissolved in 10 mL of hexane. About 1 mL of this solution was filtered through 0.2 μm PTFE filter into the HPLC vial where it was submitted to GCMS analysis.

### GCMS Analysis of Bacterial Isolates BFOA1–BFOA4 Extracts

Volatile compounds were analyzed on an Agilent 7820A gas chromatography system coupled to an Agilent 5975 series quadrupole mass spectrometer working in EI mode and resolved on a Thermo HP-5MS column (30 cm × 250 μm × 0.25 μm) (J&W Scientific, United States). One μL of the sample was injected where compounds were desorbed at a 260°C injection port. Analysis was performed in a programmed temperature: 50°C for 5 min, then (50–250°C) over 35 min using Helium as a carrier gas with a flow of 1.2 mL/min. The gas chromatography/mass spectrometry (GC/MS) interface temperature was set to 280°C. Compounds were identified using the NIST 11 library of mass spectra on Agilent ChemStation software.

### Statistical Analysis

The statistical analysis of the data was performed using analysis of variance (ANOVA) and, when significant effects were detected, the groups were compared using a *post hoc* Tukey’s HSD test. The level of significance used for all statistical tests was 5% (*p* < 0.05). The statistical program used was IBM SPSS Statistics v. 22.

## Results

### Isolation of Bacterial Isolates BFOA1–BFOA4 Antagonistic to *F. oxysporum* f. sp. *albedinis*

**FIGURE 1:**
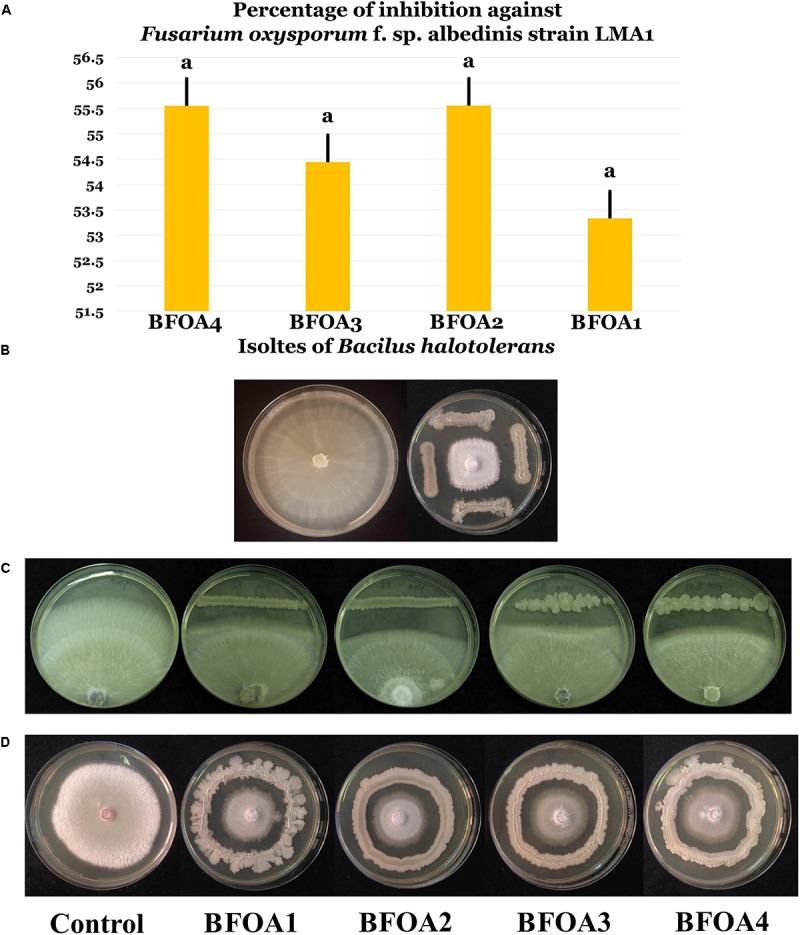
**(A)** Percentage of inhibition of four isolates (BFOA1–BFOA4) of *Bacillus halotolerans* against *Fusarium oxysporum* f. sp. *albedinis*. Data present mean ± standard error. Bars labeled with the same letter are not significantly different from each other according to Tukey’s HSD at *p* < 0.05. **(B)** Confrontation assay of antifungal activity of all four bacterial isolates against *F. oxysporum* f. sp. *albedinis*. **(C,D)** Confrontation assay of antifungal activity of the bacterial isolates against different strains of *F. oxysporum* f. sp. *albedinis.* A representative control petri dish of *F. oxysporum* f. sp. *albedinis* is presented in **(B,D)**.

Bacteria arising from two contrasting niche – endophytes of *L. monopetalum* (L.) Boiss. and rhizosphere of wheat both located in the semi-arid climate in Tunisia and Algeria, respectively – were used for the discovery of bacterial species antagonistic to *F. oxysporum* f. sp. *albedinis.* The screening conducted used more than 160 bacterial isolates and was successful in revealing four bacteria with strong antagonistic activity to *F. oxysporum* f. sp. *albedinis* LMA1 (Figures 1A–D). The bacteria proved effective in inhibiting additional strains of *F. oxysporum* f. sp. *albedinis* namely strains LMA2-LMA5 (Supplementary Figures S1, S2). The percentage of inhibition of *F. oxysporum* f. sp. *albedinis* was around 55%.

### Biocontrol Ability of Bacterial Isolates BFOA1–BFOA4 Toward the Genus *Fusarium*

**FIGURE 2:**
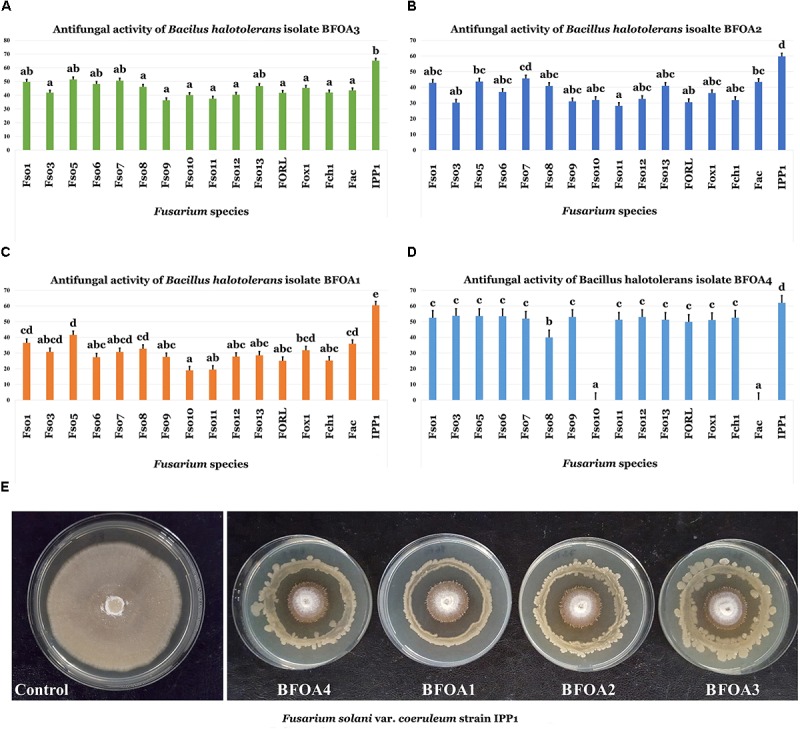
**(A–D)** Antifungal activity of four bacterial isolates of *Bacillus halotolerans* against different species of the genus *Fusarium*. Data present mean ± standard error. Bars labeled with different letters are significantly different among the treatments at *P* < 0.05 using the Tukey’s HSD test. **(E)** Confrontation assay of antifungal activity of all four bacterial isolates against *F. solani* f. sp. *coeruleum* strain IPP1. (*F. solani* isolates: Fso1, Fso3, Fso5, Fso6, Fso7, Fso8, Fso9, Fso10, Fso11, Fso12 and Fso13; *F. oxysporum* isolates: FORL and Fox1; *F. chlamydosporum* isolate: Fch1; *F. acuminatum* isolate: Fac; *F. solani* var. *coeruleum* isolate: IPP1).

Screening of bacterial isolates BFOA1–BFOA4 for the biocontrol ability of 16 *Fusarium* isolates belonging to three different species – *F. oxysporum* (with strains phytopathogenic of *Olea europaea* and tomato), *F. solani* (with different strains attacking *O. europaea* and potato), *F. acuminatum* (pathogenic on *O. europaea*) and *F. chlamydosporum* (phytopathogenic of *O. europaea*) – revealed a high efficiency of the four bacterial isolates against all phytopathogens, with the exception of BFOA4 against *F. solani* Fso10 and *F. acuminatum* Fac (Figures 2A–E).

### Effects of *Bacillus halotolerans* BFOA4 Treatment on *Fusarium oxysporum* f. sp. *radicis-lycopersici* Strain FORL Disease Severity on Tomato Fruits

BFOA4 proved effective in preventive concomitant and curative treatments in reducing tomato fruit rot severity caused by *F. oxysporum* f. sp. *radicis-lycopersici* strain FORL (Supplementary Figure S3).

### Biocontrol Ability of Bacterial Isolates BFOA1–BFOA4 Toward Other Relevant Plant Pathogens

**FIGURE 3:**
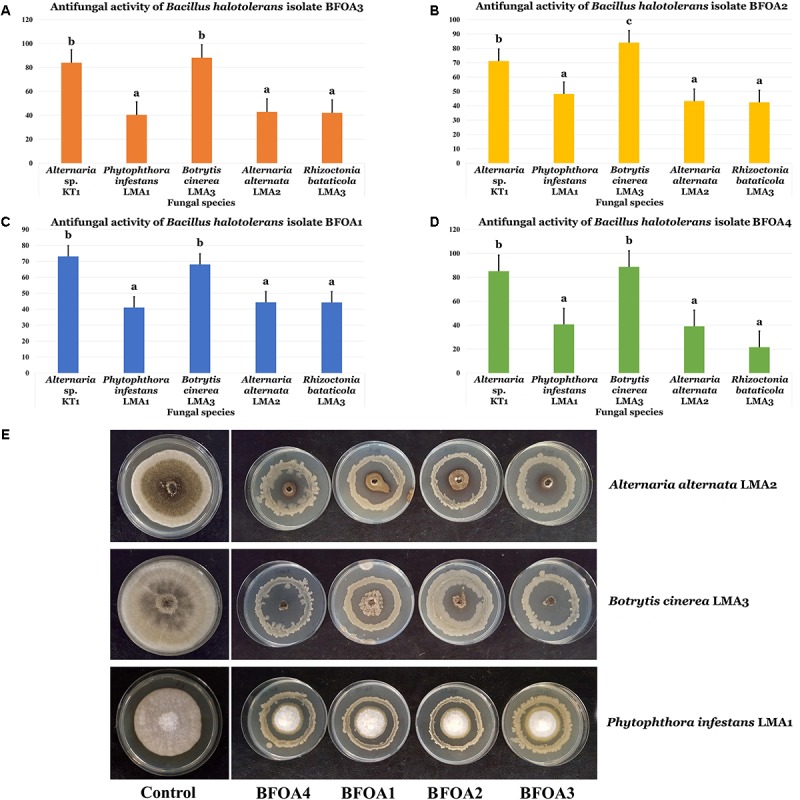
**(A–D)** Antifungal activity of four bacterial isolates of *Bacillus halotolerans* against different fungi. Data present mean ± standard error. Bars labeled with different letters are significantly different among the treatments at *P* < 0.05 using the Tukey’s HSD test. **(E)** Confrontation assay of antifungal activity of all four bacterial isolates against *Alternaria alternate* isolate LMA2, *Botrytis cinerea* isolate LMA3 *and Phytophthora infestans* isolate LMA1.

BFOA1–BFOA4 bacterial isolates also exhibited strong activities against four major phytopathogens: *Botrytis cinerea*, *Alternaria alternata*, *Phytophthora infestans*, and *Rhizoctonia bataticola* (Figures 3A–E). Isolates BFOA3 and BFOA4 proved very effective against *Alternaria* sp. KT1 and *B. cinerea* LMA3, reaching up to 100% inhibition rates in some biological repetitions of the confrontation experiments (Figure 3E).

### Abiotic Stress Tolerance of Bacterial Isolates BFOA1–BFOA4: NaCl, Temperature, PEG and pH Effects on Growth

**FIGURE 4:**
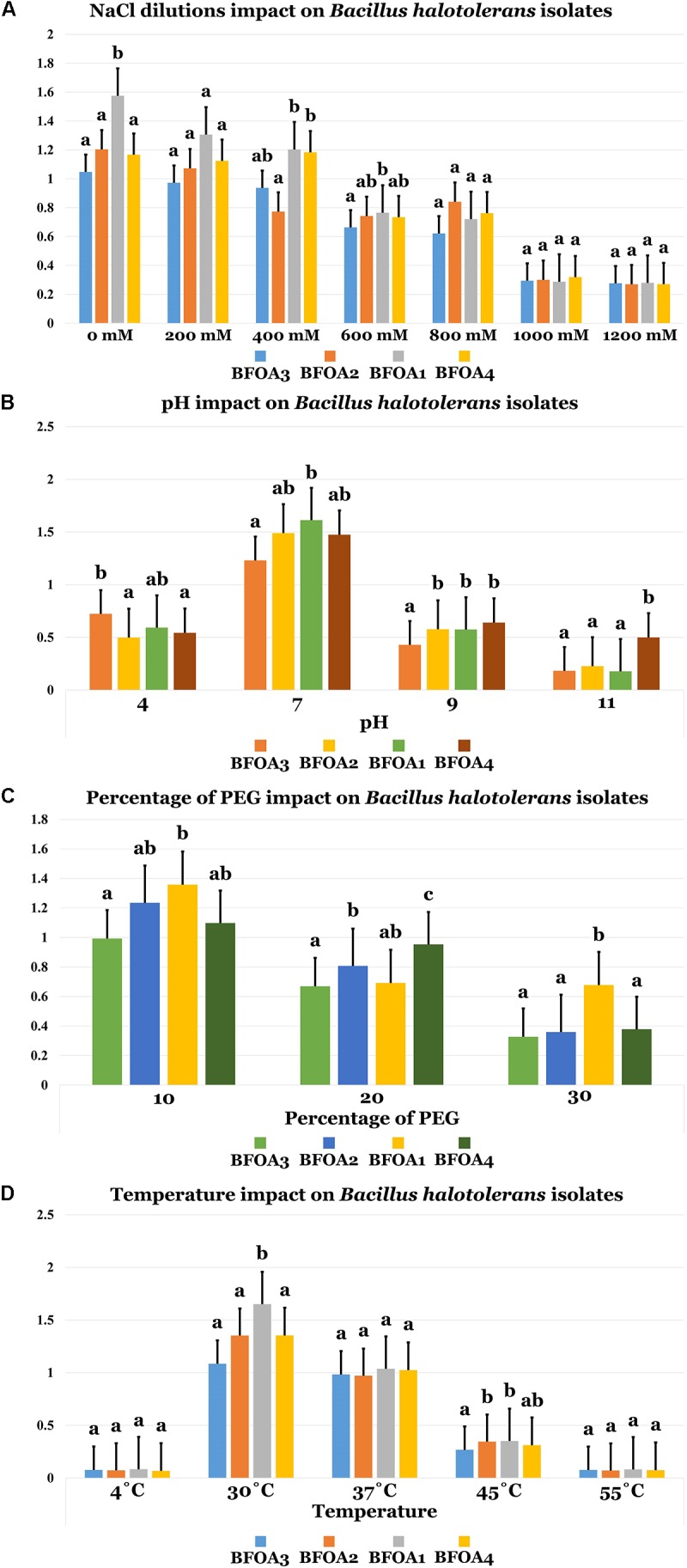
**(A)** NaCl dilutions, **(B)** pH, **(C)** percentage of PEG, and **(D)** Temperature impacts on *Bacillus halotolerans* isolaes. Data present mean ± standard error. Bars labeled with different letters are significantly different among the treatments at *P* < 0.05 using the Tukey’s HSD test.

Figure 4A unambiguously documented that NaCl concentrations up to 1M were only moderately able to interfere with BFOA1–BFOA4 growth (*p* < 0.05). Bacterial isolates BFOA1–BFOA4 had an optimal growth temperature of 30°C. However, the isolates could tolerate temperatures in the range of 4–45°C with no significant loss of growth (*p* < 0.05, Figure 4D). Bacterial isolates BFOA1–BFOA4 were also able to tolerate high PEG concentrations up to 30% with moderate loss of bacterial growth (*p* < 0.05, Figure 4C). BFOA1–BFOA4 bacterial isolates optimally grew at neutral pH 7 (Figure 4). However, they were able to tolerate pH values of 4.0 to 9.0 with moderate growth (*p* < 0.05, Figure 4B).

### PGP Potential of Bacterial Isolates BFOA1–BFOA4

**FIGURE 5:**
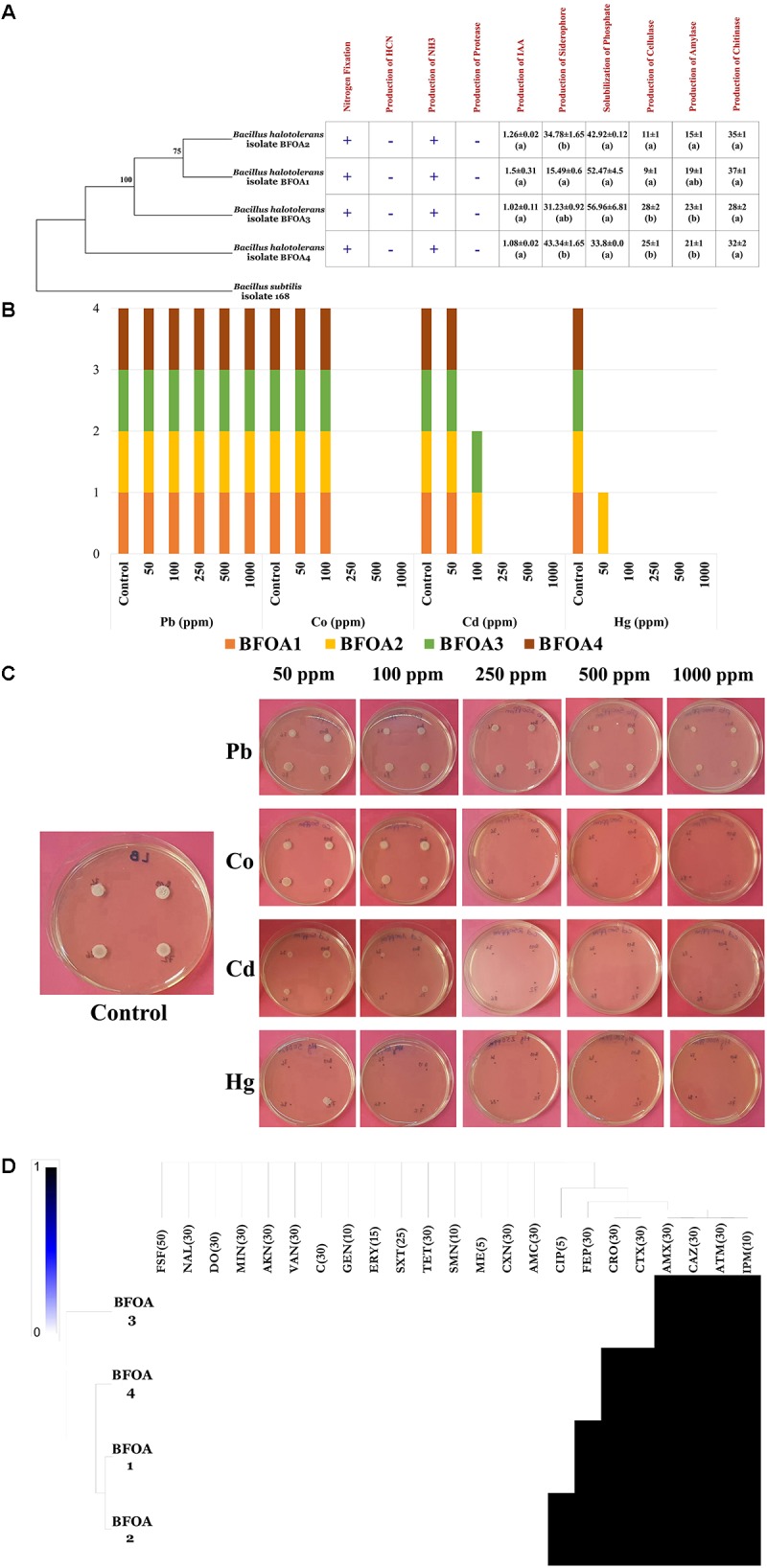
**(A)** PGP activity details of *Bacillus halotolerans* isolates. (+) and (–) represents presence and absence of the activity on each bacterial isolate. Data present mean ± standard error. Bars labeled with different letters are significantly different among the treatments at *P* < 0.05 using the Tukey’s HSD test. **(B)** Status of metal resistance and **(C)** *in vitro* assay of the bacterial isolates against different dilutions of Pb, Hg, Cd, and Co. **(D)** Heat map of antibiotic resistance of the bacterial isolates. In part **(C)** of the figure **(A)** representative petri dish of the four bacteria grown in absence of metal stress is used as control for Pb, Co; Cd, and Hg test experiments.

BFOA1–BFOA4 possessed numerous PGP activities as documented by Figure 5A. For example, they were able to fix nitrogen and produce ammonia. However, they failed to produce HCN and secrete protease activity. Auxin, siderophore, cellulase, amylase and chitinase production as well as phosphate solubilization were common features of all the bacterial isolates and reached up to 1.5 ± 0.31 μg/mL in isolate BFOA1, 43.34 ± 1.65% in strain BFOA4, 28 ± 2 mm of halo diameter in strain BFOA3, 23 ± 1 mm of halo diameter in isolate BFOA3, 35 ± 1 mm of halo diameter in isolate BFOA2 and 56.96 ± 6.81 μg/mL P_2_O_5_, respectively.

### Resistance of BFOA1–BFOA4 Bacterial Isolates to Antibiotics and Metals

Isolates BFOA1–BFOA4 showed similar trends in response to metal stress (Figure 5B). While all isolates resisted up to 1000 ppm lead and 100 ppm copper, only BFOA2 succeeded to grow at 50 ppm mercury. Isolates BFOA2 and BFOA4 were the only isolates that managed to cope with 100 ppm cadmium. With the exception of cefotaxime, ceftriaxone, cefepime and ciprofloxacin, where different susceptibility/resistance between BFOA1–BFOA4 was observed, all isolates were susceptible to fosfomycin, nalidixic acid, doxycycline, minocycline, amikacin, vancomycin, chloramphenicol, gentamycin, erythromycin, trimethoprim/sulfamethoxazole, tetracycline, streptomycin, methicillin, cephalexin, and amoxicillin/clavulanic acid and resistant to aztreonam, ceftazidime, amoxicillin, and imipenem. Only BFOA2 was resistant to ciprofloxacin. BFOA1 and BFOA2 were resistant to cefepime, while BFOA1, BFOA2, and BFOA4 were resistant to ceftriaxone and cefotaxime (Figures 5C,D).

### Identity of Bacterial Isolates BFOA1–BFOA4 and Their Phylogenomic Positions

**FIGURE 6:**
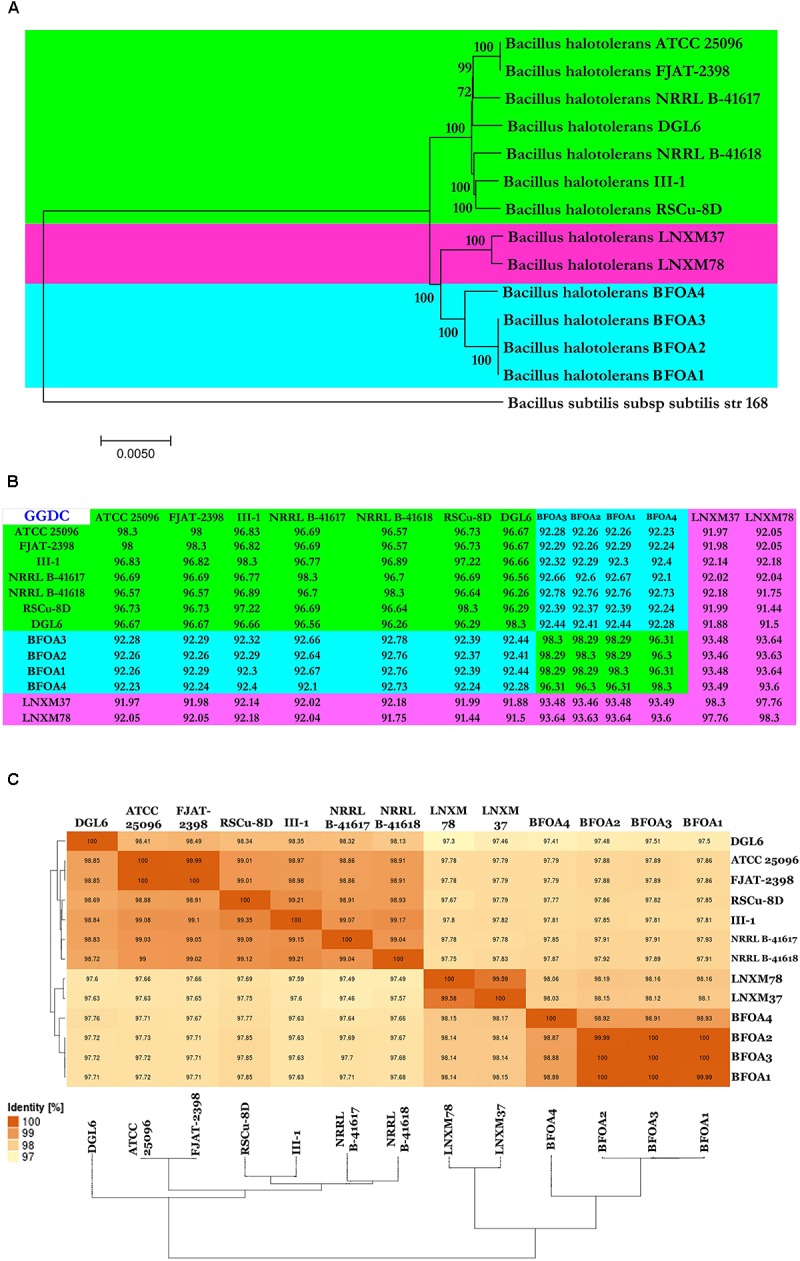
**(A)** Neighbor-joining phylogenomic tree of Gram-positive bacteria *Bacillus halotolerans* isolates. *Bacillus subtilis* subsp. *subtilis* isolate 168 was used as outgroup. Supports for branches were assessed by bootstrap resampling of the data set with 1000 replications. **(B,C)** Genome-to-Genome Distance Calculation (GGDC) and Average nucleotide identity (ANI) values between each indicated isolate were calculated with GGDC 2 and EzBiocloud web-based programs and showed 3 species candidates based on 70 and 95% similarity thresholds.

Based on genomic analysis, all the bacterial isolates, BFOA1–BFOA4, were identified as *B. halotolerans*. Both Genome-to-Genome Distance (GGD) analysis and Average Nucleotide Identity (ANI) analysis revealed that isolates BFOA1–BFOA4 represent isolates of the species *B. halotolerans*. Genomes of the 9 isolates of *B. halotolerans* available in GenBank along with the four genomes of isolates BFOA1–BFOA4 generated in this study were selected to perform phylogenomic analysis of the *B. halotolerans* isolates and ascertain the phylogenetic position of BFOA1–BFOA4 (Supplementary Table S1). Within *B. halotolerans*, genome size ranged from 3.74 to 4.4 Mbp (Supplementary Table S1). GC content, however, was between 43 and 44%. Genome-to-Genome Distance (GGD) analysis revealed that all analyzed isolates represented the same species sensu Meier-Kolthoff et al. (2013), where 70% similarity between two genomes was established as a suitable cut-off and the gold standard threshold for species boundaries (Figure 6B). Average Nucleotide Identity (ANI) analysis also revealed a single species sensu Richter and Rossello-Mora (2009), where a 95–96% cut-off was set up to delimit species boundaries and confirmed, therefore, the observations drawn using GGD analysis (Figure 6C). Whole genome phylogeny also confirmed earlier results and clearly documented the limping of the 13 isolates in the species *B. halotolerans* (Figure 6A).

### Characterization of the Core and the Pan Genome of *B. halotolerans*

**FIGURE 7:**
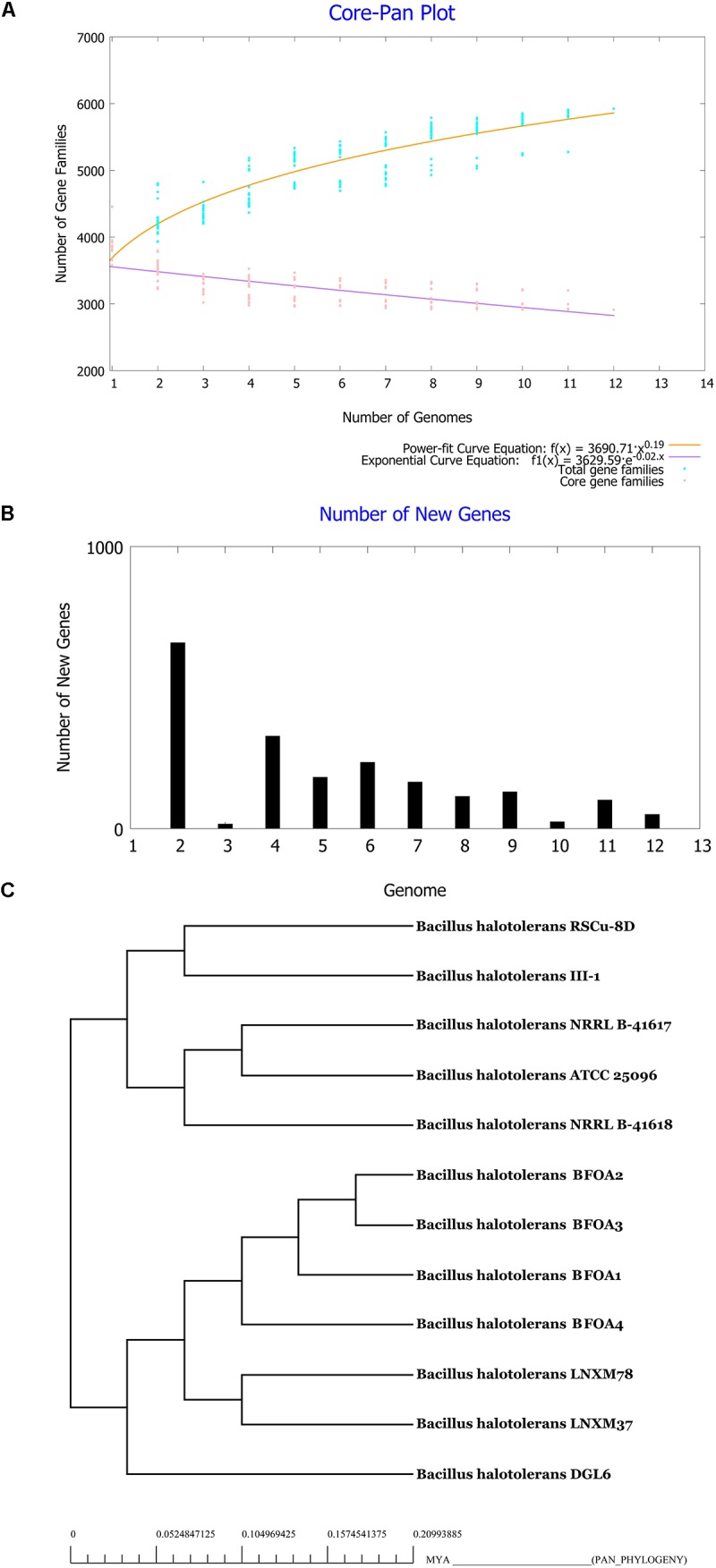
**(A)** Core-Pan genome plot of *Bacillus halotolerans* isolates based on number of genomes and number of gene families. **(B)** Number of new genes identified in genomes of the bacteria. **(C)** Tree of Pan-genome related phylogeny of *Bacillus halotolerans* isolates.

Pan and core genome analysis is required to explore full genomic and metabolic potentialities of a species (Belbahri et al., 2017). Pan and core genomes of *B. halotolerans* were then conducted using the genomes of the 13 isolates available and are presented in Figures 7A,B. Figure 7A shows clearly that the pan-genome of *B. halotolerans* increased following the increase in the number of genomes analyzed. According to Heaps’ law as implemented in Tettelin et al. (2008), the pan-genome of *B. halotolerans* species increased with α values of 0.19, suggesting that it might be an open pan-genome.

Genome-based phylogenetic relationships among the 13 genomes of *B. halotolerans* isolates were inferred using the concatenated amino acid sequences of core-genome derived from the 13 genomes. The phylogenetic tree was in agreement with the results obtained using GGD and ANI analysis suggesting that all isolates belong to *B. halotolerans* (Figure 7C).

### Functional Characterization of the Core, Accessory and Unique Genomes of *B. halotolerans*

**FIGURE 8:**
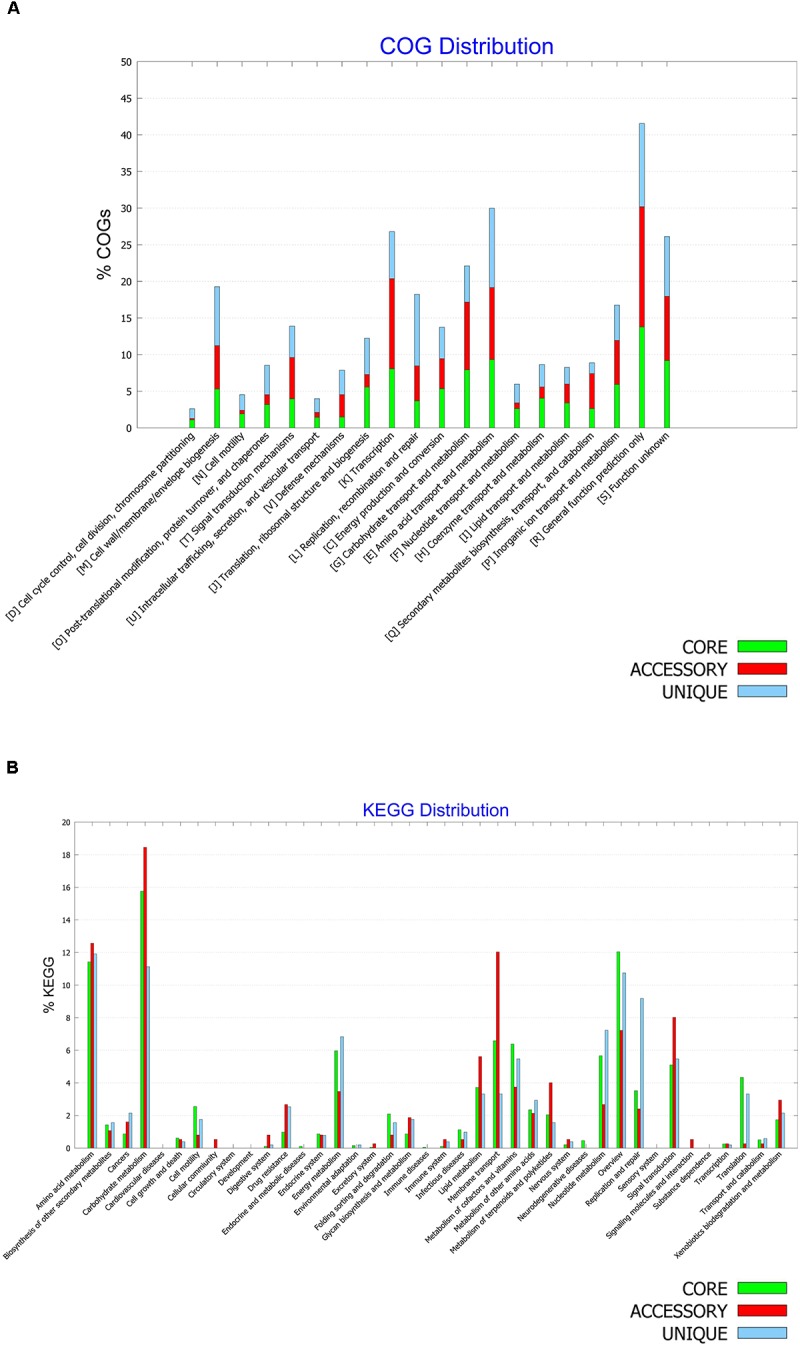
**(A)** COG and **(B)** KEGG distribution among core, accessory and unique genomes of *Bacillus halotolerans* isolates.

A pan-genome is believed to represent full potentialities of a given species, while a core genome is considered to document common genes shared by all genomes of the given species and therefore describes common metabolic and functional features of the species (Belbahri et al., 2017). The COG distributions of pan- and core- genomes of *B. halotolerans* were generated and compared (Figures 8A,B).

The COG distributions of the core, accessory and unique genomes of the species are presented in Figure 8A and showed clear differences between the three genomes. While functional genes related to defense mechanisms (V) were enriched in the unique genome, secondary metabolite biosynthesis, transport, and catabolism (Q) were enriched in the accessory genome, and the coenzyme transport and metabolism (H) as well as energy production and conversion (C) were enriched in the core genome.

The metabolic features of *B. halotolerans* were also investigated through a KEGG pathway analysis (Figure 8B). Overall KEGG metabolic pathways distribution among the three genomes showed that metabolism of terpenoids and polyketides, xenobiotics degradation as well as carbohydrate, lipid and amino acid metabolism along with membrane transport and signal transduction, were enriched in the accessory genome. Nucleotide and energy metabolism along with replication and repair were enriched in the unique genome. Metabolism of cofactors and vitamins, cell motility and translation were particularly enriched in the core genome of *B. halotolerans*.

### Genome Mining of All PGP Genes of Available *Bacillus halotolerans* Genomes

**FIGURE 9:**
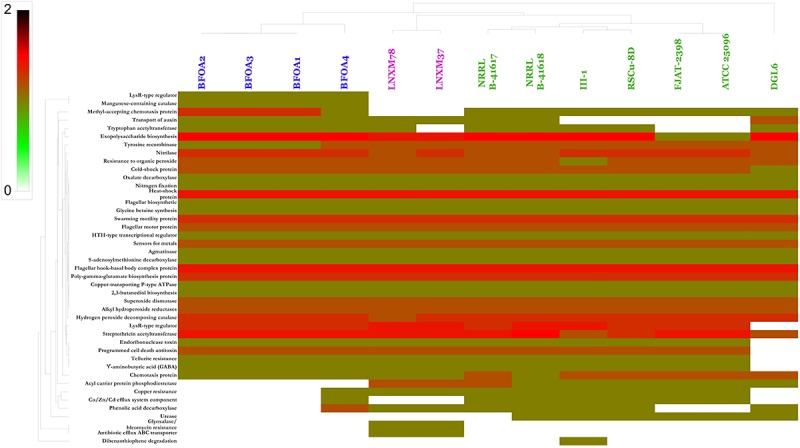
Heat map resulting from the genome mining of genes contributing to plant-beneficial functions in *Bacillus halotolerans* isolates. Bacterial isolates belonging to the same species are highlighted with the same colors.

Genome mining of PGP activities of *B. halotolerans* isolates was conducted using a homology-based mining strategy of genes contributing to plant-beneficial functions. Targeted plant beneficial functions-related genes were described in the experimental section and are classified into the following: (i) genes contributing to nutrient acquisition, (ii) genes conferring PGPR fitness, (iii) genes conferring root colonization and growth promotion factors, (iv) plant growth promoting traits (hormones), (v) plant protection from oxidative stress, (vi) plant induction of disease resistance, (vii) antibiotics and related compounds, (viii) resistance to drugs and heavy metals, and (ix) degradation of aromatic compounds (Cherif-Silini et al., 2012; Belbahri et al., 2017). Results suggested that all the isolates, with minor exceptions, were endowed with a large number of PGP capacities mined (Figure 9). *B. halotolerans* isolates BFOA1–BFOA4 showed the presence of mined genes independently of their site of collection.

### Secondary Metabolite Biosynthesis Abilities of the Pan, Core and Accessory Genomes of *B. halotolerans*

**FIGURE 10:**
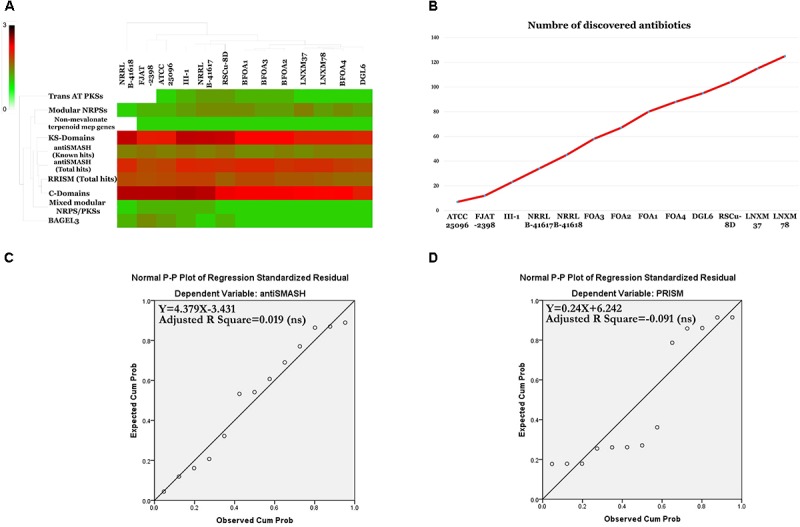
**(A)** Heat map resulting from the genome mining of genes contributing to secondary metabolite clusters. **(B)** Number of discovered secondary metabolites. **(C)** Statistically non-significant linear relationship between genome sizes and antiSMASH total hits (*p* < 0.05). **(D)** Statistically non-significant linear relationship between genome sizes and PRISM total hits.

The programs antiSMASH 3.0 (Weber et al., 2015), prediction informatics for secondary metabolomes PRISM (Skinnider et al., 2015), NapDos (Ziemert et al., 2012), NP.search (Li et al., 2009), and the bacteriocin specific software BAGEL3 (Van Heel et al., 2013) were used to uncover the secondary metabolite clusters presented in the genomes of the *B. halotolerans* BFOA1–BFOA4 isolates as well as in the other 9 genomes available for different *B. halotolerans* isolates (Supplementary Table S1). Results presented in Figure 10 and Supplementary Table S3 highlighted numerous diverse clusters with all used programs. Numerous secondary metabolite clusters encode unknown yet-to-be-discovered secondary metabolites, while some clusters encode known products such as surfactin, bacillibactin, subtilosin A, bacilysin, bacillaene, and fengycin. Rarefaction analysis of secondary metabolite clusters resulting from the genome sequencing of the *B. halotolerans* collection (13 isolates) clearly highlighted that saturation was far from being reached (Figure 10B). The correlation between genome size and number of gene clusters known to be involved in secondary products biosynthesis generated using antiSMASH and PRISM were analyzed and clearly showed no good correlation in either case (Figures 10C,D).

### Predicted Natural Products Richness and Location Within *B. halotolerans* Genomes

**FIGURE 11:**
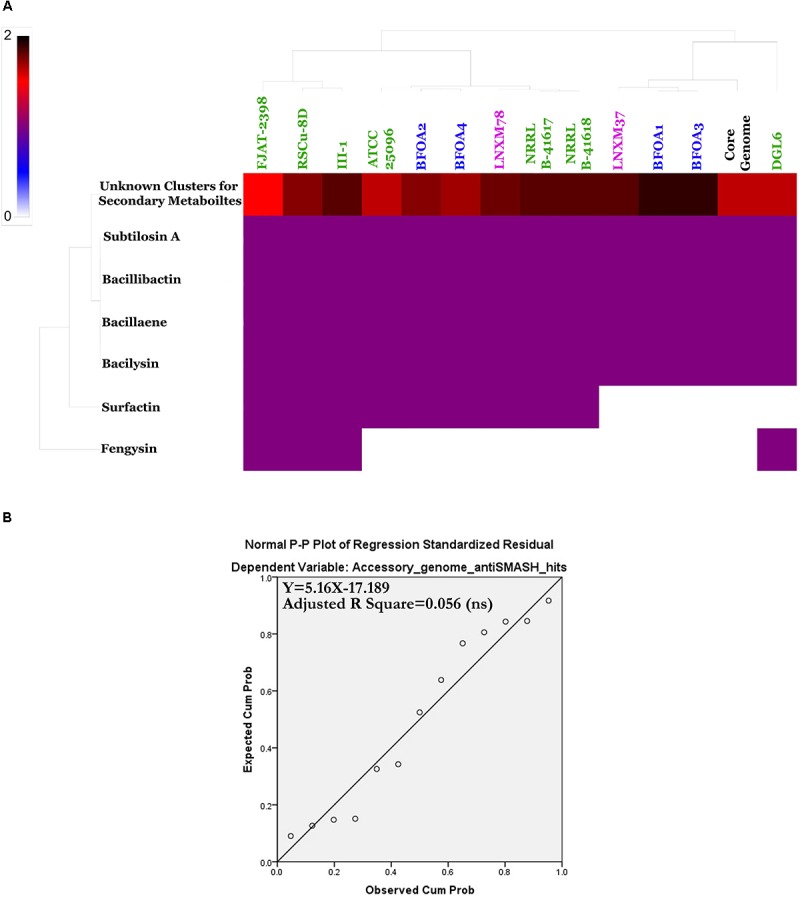
**(A)** Heat map of *Bacillus halotolerans* accessory genome secondary metabolites. **(B)** Non-significant linear relationship between genome sizes and accessory genome antiSMASH total hits (*p* < 0.05).

A natural products survey in the core genome and the accessory genomes of the *B. halotolerans* isolates highlighted the high diversity and numbers of unknown secondary metabolites thriving in the genomes of the collection (Figure 10A and Supplementary Table S3). Subtilosin A, bacillibactin, bacillaene and bacilysin were located within the core genome. All remaining secondary clusters, encoding unknown secondary metabolites for the majority of them, were harbored by the accessory genome of the collection (Figure 11A). A weak correlation between the size of the accessory genome and the number of secondary products encoding gene clusters generated using antiSMASH (Figure 11B) was recognized. Only 5.6% of the variance in the number of secondary metabolite clusters could be explained by genome size (Figure 11B).

### LCMS and GCMS Analysis of *B. halotolerans* Isolates BFOA1–BFOA4 Extracts

#### LCMS Analysis

LC-HRMS analysis of the secondary metabolites secretome of the *B. halotolerans* isolates BFOA1–BFOA4 revealed a rich arsenal with documented activities in PGP, biocontrol/antimicrobial activity or herbicidal/insecticidal activities. Indole propionic acid and rotihibin-B for example were reported with PGP activities (Pain and Roy, 1981; Fukuchi et al., 1995). Inthomycin-A, cyclo(L-Val-L-Phe), 5-deoxybutirosamine, plipastatin A1 (Taylor and Schmitz, 1976; Webb et al., 2008; Guo et al., 2011; Ma and Hu, 2018) were reported to have biocontrol/antimicrobial activity. Inthomycin-A was also reported to have herbicidal activity (Webb et al., 2008). Additionally, numerous compounds with no reported biological activity have also been detected (Supplementary Table S4).

#### GCMS Analysis

The GCMS analysis of volatile organic compounds (VOCs) from *B. halotolerans* isolates BFOA1–BFOA4 revealed the presence of up to 38 VOCs in the volatile extract fractions of the different bacteria isolates (Supplementary Table S5). While the majority of the VOCs did not have any reported biological activity connected with PGP, biocontrol/antimicrobial activity or herbicidal/insecticide activity, we successfully detected pulegone reported as having strong insecticide activity (Franzios et al., 1997), 2-undecanone reported to be a strong insect repellent (Stephen et al., 2002) and germacrene D reported to have antimicrobial and insecticidal properties (Adio, 2009).

## Discussion

The screening conducted in this study aimed to isolate bacterial species antagonistic to *F. oxysporum* f. sp. *albedinis.* Therefore, two locations in Tunisia and Algeria within semi-arid climates have been targeted by endophyte and PGPR isolation from *L. monopetalum* (L.) Boiss. and rhizosphere of wheat, respectively. These two sites were selected to fit the definable environmental limits for the existence of the biological control agent (Debbi et al., 2018; Gomez-Lama Cabanas et al., 2018; Guardado-Valdivia et al., 2018). More than 140 bacterial isolates were recovered from these two sites and screened for their inhibitory effect against *F. oxysporum* f. sp. *albedinis* using *in vitro* confrontation assays. The screening yielded four bacterial isolates with strong activity against *F. oxysporum* f. sp. *albedinis*. Screening of these bacterial isolates against other 16 *Fusarium* isolates belonging to three species – *F. oxysporum* (with strains pathogenic on olive and tomato), *F. solani* (with different strains attacking olive and potato), *F. acuminatum* (pathogenic on olive) and *F. chlamydosporum* (phytopathogenic of *O. europaea*) – allowed us to prove their inhibitory activity against all the collection, except BFOA4, which was not active against *F. solani* Fso10 and *F. acuminatum* Fac. These results indicated that BFOA1–BFOA4 are active against the genus *Fusarium*. Therefore, they could be considered as plant wardens against resilient *Fusarium* sensu Bernal et al. (2017), who suggested a similar status for *Pseudomonas putida* T6SS. This outcome is crucial for the targeted ecosystem, where *Fusarium* is a major threat targeting approximately all plant types in the oasis. In fact, the oasis cultivation system is based on a three-stage canopy level system. The highest tier includes the date palm, while the middle tier is reserved for arboriculture and the lowest tier for annual/pluriannual crops. The genus *Fusarium* is able to infect date palms, olive trees and annual/pluriannual crops such as tomato and potato among other plants. It is clearly established that the *Fusarium* spp. infecting annual/pluriannual crops planted in the lower tier of the oasis was isolated from surrounding olive trees (Trabelsi et al., 2017). BFOA4 tested on preventive concomitant and curative treatments in reducing tomato fruit rot severity caused by *F. oxysporum* f. sp. *radicis-lycopersici* strain FORL. This suggests the ability of *Bacillus halotolerans* to exert its biocontrol effect *in planta*.

Additionally, the bacterial isolates exhibited strong activities against four other major phytopathogens occurring in the oasis ecosystems targeted in the study; *B. cinerea*, *A. alternata*, *Phytophthora infestans*, and *R. bataticola*. This finding reinforces the utility of the biocontrol strains isolated to suppress pathogens occurring in the oasis ecosystem.

To understand their physico-chemical requirements for growth, we have examined the effect of temperature, pH, NaCl and drought simulated using varying PEG concentrations. Our results indicated that isolates BFOA1–BFOA4 had the ability to grow at temperatures up to 45°C, pH range of 5 to 10, and tolerated high concentrations of NaCl and up to 30% PEG. These characteristics made them suitable biocontrol agents for the considered oasis ecosystems. They can support the high temperatures and drought conditions (El Hassni et al., 2007; Dihazi et al., 2012; Jiang et al., 2017) as well as high salinity (Seydehmet et al., 2018) prevalent in these ecosystems. Since the isolates are either endophytes or rhizosphere inhabitants, direct and indirect PGP features including growth on nitrogen-free medium, phosphate solubilization and auxin biosynthesis, as well as resistance to metal and xenobiotic stress were assessed. Results unambiguously documented the strong PGP activity of BFOA1–BFOA4. All isolates showed nitrogen fixation and NH_3_ production abilities and failed to produce HCN or secrete protease activity. They showed auxin, siderophore, cellulase, amylase and chitinase production as well as phosphate solubilization, which were common features of all bacterial isolates. These PGP abilities are characteristic of endophytes and rhizospheric bacteria (Belbahri et al., 2017; Mefteh et al., 2017). Isolates BFOA1–BFOA4 also showed similar trends and high resistance in response to metal stress. This feature is not surprising given the high metal levels in irrigation water used in oasis ecosystems (Aly et al., 2014; Khezzani and Bouchemal, 2018). This feature is highly suitable in PGP bacteria and helps to alleviate the effects of phyto-toxic metals on plant growth and productivity, which promotes the sustainability of the oasis ecosystems (Pramanik et al., 2018). BFOA1–BFOA4 also proved susceptible to the antibiotics fosfomycin, nalidixic acid, doxycycline, minocycline, amikacin, vancomycin, chloramphenicol, gentamycin, erythromycin, trimethoprim/sulfamethoxazole, tetracycline, streptomycin, methicillin, cephalexin and amoxicillin/clavulanic acid and resistant to aztreonam, ceftazidime, amoxicillin, and imipenem. BFOA1 and BFOA2 were resistant to cefepime, while BFOA1, BFOA2, and BFOA4 were resistant to ceftriaxone and cefotaxime. Co-occurrence of antibiotic and metal resistance genes was also revealed by Li et al. (2017) in a large set of complete genome collection.

All the features reported for BFOA1–BFOA4 prompted us to investigate their phylogenetic affinities. While 16S sequencing proved poor in allowing exact affiliation of the isolates (data not shown), genome sequencing was highly effective. BFOA1–BFOA4 belonged to *B. halotolerans* as documented by gold standards for bacterial classification (ANI, GGD and phylogenomic approaches). Comparative genomics of *B. halotolerans* isolates available to date allowed us to show that the pan-genome of the species might be an open pan-genome experiencing frequent evolutionary changes through gene gains and losses or lateral gene transfers for efficient environmental adaptations. Similar results have been obtained for sister species such as *B. amyloliquefaciens* (Belbahri et al., 2017). Analysis of the pan, accessory and unique genomes of the different isolates showed clearly high enrichment of PGP abilities and secondary metabolite gene clusters in the core genome with at least four secondary metabolite clusters (subtilosin A, bacillibactin, bacillaene, and bacilysin). This feature could be related to the *B. halotolerans* life style in association with plant rhizosphere and roots as suggested for *B. amyloliquefaciens* by Belbahri et al. (2017). Analysis of secondary metabolite secretome of the *B. halotolerans* BFOA1–BFOA4 isolates by LC-HRMS analysis revealed a rich arsenal with documented activities in PGP, biocontrol/antimicrobial or herbicidal/insecticide activities. Indole propionic acid and rotihibin-B for example were reported to have PGP activities (Pain and Roy, 1981; Fukuchi et al., 1995). Inthomycin-A, cyclo(L-Val-L-Phe), 5-deoxybutirosamine, and plipastatin A1 (Taylor and Schmitz, 1976; Webb et al., 2008; Guo et al., 2011; Ma and Hu, 2018) were reported to have biocontrol/antimicrobial activities. Similarly, GCMS analysis of VOCs from *B. halotolerans* BFOA1–BFOA4 revealed the presence of up to 38 VOCs in the volatile extract fractions of the different bacteria. Pulegone was reported as a strong insecticide (Franzios et al., 1997), 2-undecanone reported to be a strong insect repellent (Stephen et al., 2002) and germacrene D reported to have antimicrobial and insecticidal properties (Adio, 2009) were detected in the GCMS analysis. In conclusion, our study aimed to provide biological control of the Bayoud disease of date palm groves by *F. oxysporum* f. sp. *albedinis* and will definitely be successful in reaching this objective and in providing a plant warden for resistance against *Fusarium*-resilient pathogens that have dramatic effects on plant productivity in oasis ecosystems, once the results will be validated in Greenhouse and field tests. Future studies will target the possibility of developing novel *B. halotolerans*-based bio-formulations having PGPR properties for enhanced production of agricultural crops and coping with *Fusarium* attacks in oasis production systems.

## Ethics Statement

This research did not involve any work with human participants or animals by any of the authors.

## Author Contributions

HC-S and LB conceived and designed the experiments. HS, HC-S, LL, MQ, AV, MR, and LB performed the experiments. HC-S, LB, HS, ACB, and MR analyzed the data. LB, HC-S, AS, BY, LL, MT, TO, and FA contributed reagents, materials, and analysis tools. LB, HS, ACB, LL, MR, TO, and MT wrote and enriched the literature.

## Conflict of Interest Statement

The authors declare that the research was conducted in the absence of any commercial or financial relationships that could be construed as a potential conflict of interest.

## Footnotes

1 http://www.ige3.unige.ch/genomics-platform.php

2 http://www.ezbiocloud.net/tools/ani

3 http://ggdc.dsmz.de/distcalc2.php

4 http://realphy.unibas.ch

5 http://www.kegg.jp/blastkoala
