# Supplementary material for: Screening for Fusarium Antagonistic Bacteria From Contrasting Niches Designated the Endophyte Bacillus halotolerans as Plant Warden Against Fusarium
Source: Front Microbiol. 2019 Jan 11;9:3236. doi: 10.3389/fmicb.2018.03236 (PMC6336696; doi:10.3389/fmicb.2018.03236)
Supplement: Supplementary file 1 [file Data_Sheet_1.pdf]

Supplementary Table S1. Details of *Bacillus halotolerans* isolates used in this study.

| Species | Strain | Genome size (Mb) | Plasmid | Description | GenBank Accession |
|---|---|---|---|---|---|
| ***Bacillus halotolerans*** | FJAT-2398 | 4.09077 | - | Isolated from agricultural soils (Germany) | LUUT00000000.1 |
| | ATCC 25096 | 4.07273 | - | Isolated from desert soils (Morocco) | LPVF00000000.1 |
| | NRRL B-41617 | 4.09146 | - | Isolated from river (Spain) | LPVD00000000.1 |
| | III-1 | 4.13992 | - | Isolated from roots of *Phoenix dactylifera* (Tunisia) | MBQV00000000.1 |
| | BFOA2 | 4.44148 | - | Isolated from roots of *Limoniastrum monopetalum* (Tunisia) | PVXB00000000.1 |
| | BFOA4 | 4.13265 | - | Isolated from rhizosphere of wheat plant (Algeria) | PVXD00000000.1 |
| | BFOA1 | 4.09488 | - | Isolated from roots of *Limoniastrum monopetalum* (Tunisia) | PVXA00000000.1 |
| | BFOA3 | 4.15264 | - | Isolated from roots of *Limoniastrum monopetalum* (Tunisia) | PVXC00000000.1 |
| | NRRL B-41618 | 4.20528 | - | Isolated from river (Spain) | LPVE00000000.1 |
| | RSCu-8D | 4.15979 | - | Isolated from *Cuminum cyminum* (India) | NERK00000000.1 |
| | LNXM37 | 4.08166 | - | Isolated from soil (environmental sample, China) | PVQP00000000.1 |
| | LNXM78 | 4.17021 | - | Isolated from soil (environmental sample, China) | PVQQ00000000.1 |
| | DGL6 | 3.73909 | - | Isolated from a salt lake (China) | PVWC00000000.1 |

Supplementary Table S2. Details of fungal species used in this study.

| | Species | Strain | Source | Country | GenBank Accession |
|---|---|---|---|---|---|
| | | | | | ITS |
| 1 | *Fusarium solani* | Fso3 | *Olea europaea* | Tunisia | KU528851 |
| 2 | *Fusarium solani* | Fso12 | *Olea europaea* | Tunisia | KU528862 |
| 3 | *Fusarium solani* | Fso11 | *Olea europaea* | Tunisia | KU528861 |
| 4 | *Fusarium solani* | Fso7 | *Olea europaea* | Tunisia | KU528857 |
| 5 | *Fusarium solani* | Fso5 | *Olea europaea* | Tunisia | KU528854 |
| 6 | *Fusarium solani* | Fso6 | *Olea europaea* | Tunisia | KU528855 |
| 7 | *Fusarium solani* | Fso8 | *Olea europaea* | Tunisia | KU528858 |
| 8 | *Fusarium solani* | Fso9 | *Olea europaea* | Tunisia | KU528859 |
| 9 | *Fusarium solani* | Fso1 | *Olea europaea* | Tunisia | KU528848 |
| 10 | *Fusarium solani* | Fso10 | *Olea europaea* | Tunisia | KU528860 |
| 11 | *Fusarium solani* | Fso13 | *Olea europaea* | Tunisia | KU528863 |
| 12 | *Fusarium solani* | Fso2 | *Olea europaea* | Tunisia | KU528850 |
| 13 | *Fusarium oxysporum* | Fox1 | *Olea europaea* | Tunisia | KU528844 |
| 14 | *Fusarium oxysporum* f.sp. *radices lycopersicum* | FORL | Tomato | Tunisia | - |
| 15 | *Fusarium acuminatum* | Fac | *Olea europaea* | Tunisia | KU528866 |
| 16 | *Fusarium chlamydosporum* | Fch1 | *Olea europaea* | Tunisia | KU528845 |
| 17 | *Alternaria* sp. | KT1 | *Pistacia vera* | Tunisia | - |
| 18 | *Rhizoctonia bataticola* | MAT1 | *Olea europaea* | Tunisia | - |
| 19 | *Phytophthora infestans* | LMA1 | Potato | Algeria | - |
| 20 | *Alternaria alternata* | LMA2 | Tomato | Algeria | - |
| 21 | *Botrytis cinerea* | LMA3 | Strawberry | Algeria | - |
| 22 | *Fusarium oxysporum* f.sp. *albedinis* | LMA1 | *Phoenix dactylifera* | Algeria | - |
| 23 | *Fusarium oxysporum* f.sp. *albedinis* | LMA2 | *Phoenix dactylifera* | Algeria | - |
| 24 | *Fusarium oxysporum* f.sp. *albedinis* | LMA3 | *Phoenix dactylifera* | Algeria | - |
| 25 | *Fusarium oxysporum* f.sp. *albedinis* | LMA4 | *Phoenix dactylifera* | Algeria | - |
| | *Fusarium oxysporum* f.sp. *albedinis* | LMA5 | *Phoenix dactylifera* | Algeria | - |
| 23 | *Fusarium solani* var. *coeruleum* | IPP1 | Potato | France | - |

Supplementary Table S3. Secondary metabolites of *Bacillus halotolerans* isolates identified with antiSMASH program.

| Strain clusters | Type | Length (bp) | Most similar known clusters | Predicted core clusters |
|---|---|---|---|---|
| **ATCC 25096** | | | | |
| Cluster 1 | Nrps | 65255 | Surfactin biosynthetic gene cluster (86% of genes show similarity) | 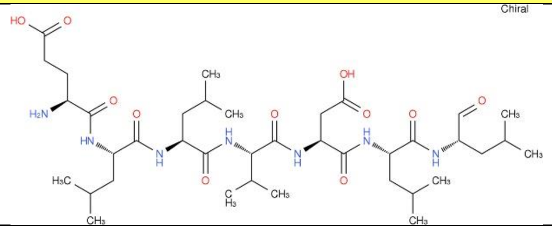 |
| Cluster 2 | Nrps | 2695 | - | - |
| Cluster 3 | Nrps | 12795 | Plipastatin biosynthetic gene cluster (38% of genes show similarity) | 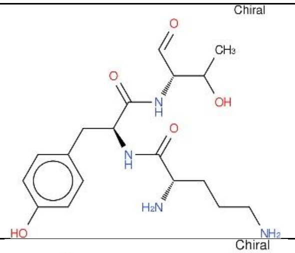 |
| Cluster 4 | Nrps | 49738 | Bacillibactin biosynthetic gene cluster (100% of genes show similarity) | 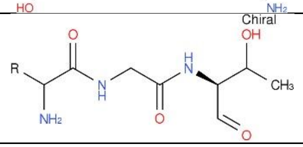 |
| Cluster 5 | Sactipeptide-Head to tail | 21612 | Subtilosin A biosynthetic gene cluster (100% of genes show similarity) | - |
| Cluster 6 | Other | 41422 | Bacilysin biosynthetic gene cluster (100% of genes show similarity) | - |
| Cluster 7 | Nrps | 90306 | Fengycin biosynthetic gene cluster (80% of genes show similarity) | 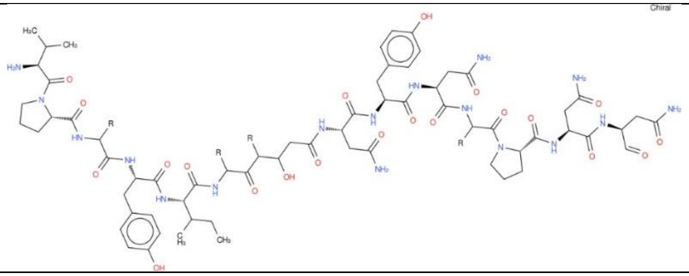 |

Supplementary Table S3. Continued.

| Strain clusters | Type | Length (bp) | Most similar known clusters | Predicted core clusters |
|---|---|---|---|---|
| **ATCC 25096** | | | | |
| Cluster 8 | Transatpks-Otherks-Nrps | 110097 | Bacillaene biosynthetic gene cluster (100% of genes show similarity) | 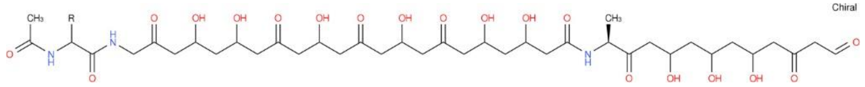 |
| Cluster 9 | Terpene | 20806 | - | - |
| Cluster 10 | Terpene | 21898 | - | - |
| Cluster 11 | T3pks | 41097 | - | - |
| Cluster 12 | Lantipeptide | 18699 | - | - |

Supplementary Table S3. Continued.

| Strain clusters | Type | Length (bp) | Most similar known clusters | Predicted core clusters |
|---|---|---|---|---|
| **FJAT-2398** | | | | |
| Cluster 1 | T3pks | 41097 | - | - |
| Cluster 2 | Terpene | 21898 | - | - |
| Cluster 3 | Transatpks-Nrps | 114372 | Fengycin biosynthetic gene cluster (93% of genes show similarity) | 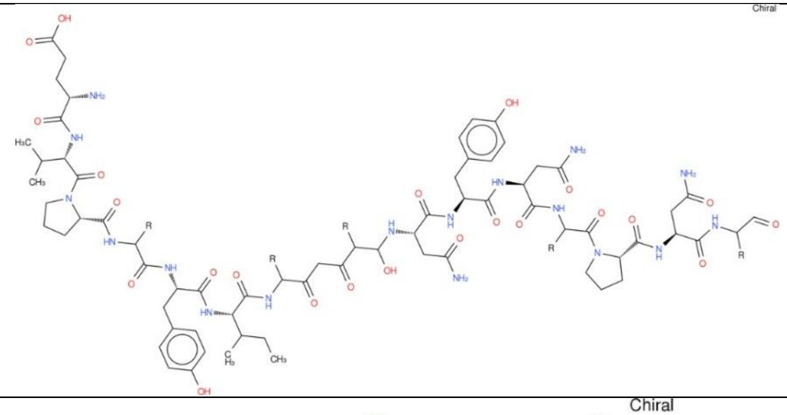  |
| Cluster 4 | Transatpks-Otherks-Nrps | 110096 | Bacillaene biosynthetic gene cluster (100% of genes show similarity) | 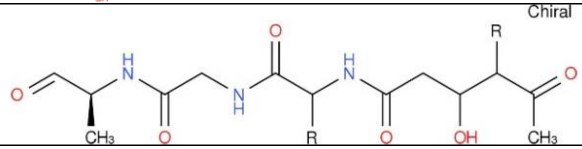  |
| Cluster 5 | Terpene | 20806 | - | - |
| Cluster 6 | Nrps | 65255 | Surfactin biosynthetic gene cluster (86% of genes show similarity) | 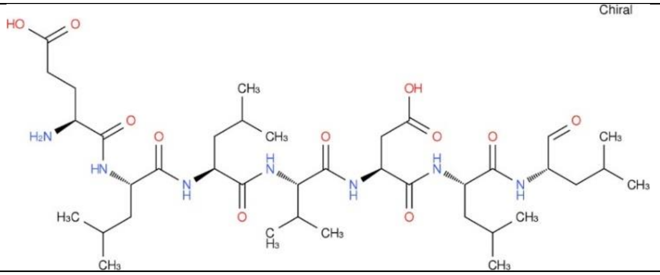  |
| Cluster 7 | Lantipeptide | 18464 | - | - |
| Cluster 8 | Nrps | 49738 | Bacillibactin biosynthetic gene cluster (100% of genes show similarity) | 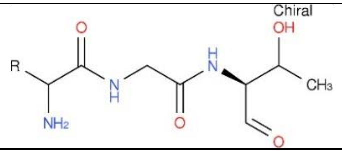  |

Supplementary Table S3. Continued.

| Strain clusters | Type | Length (bp) | Most similar known clusters | Predicted core clusters |
|---|---|---|---|---|
| **FJAT-2398** | | | | |
| Cluster 9 | Sactipeptide-Head to tail | 21612 | Subtilosin A biosynthetic gene cluster (100% of genes show similarity) | - |
| Cluster 10 | Other | 41422 | Bacilysin biosynthetic gene cluster (100% of genes show similarity) | - |
| Cluster 11 | Nrps | 12929 | Plipastatin biosynthetic gene cluster (38% of genes show similarity) | 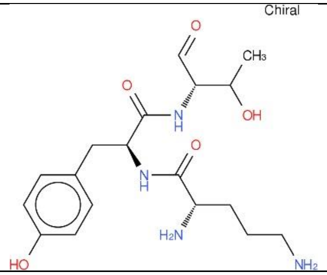 |

Supplementary Table S3. Continued.

| Strain clusters | Type | Length (bp) | Most similar known clusters | Predicted core clusters |
|---|---|---|---|---|
| **III-1** | | | | |
| Cluster 1 | Nrps | 14107 | Fengycin biosynthetic gene cluster (26% of genes show similarity) | 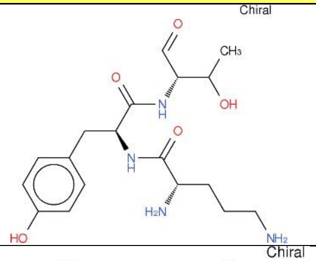 |
| Cluster 2 | Nrps | 10652 | Plipastatin biosynthetic gene cluster (23% of genes show similarity) | 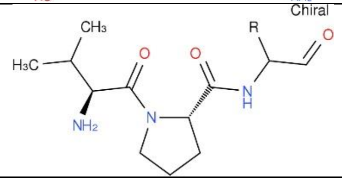 |
| Cluster 3 | Transatpks | 8404 | Elansolid biosynthetic gene cluster (10% of genes show similarity) | 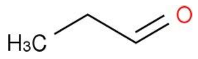 |
| Cluster 4 | Transatpks | 5353 | - | - |
| Cluster 5 | Transatpks | 2123 | - | - |
| Cluster 6 | Terpene | 20806 | - | - |
| Cluster 7 | Nrps-Transatpks-Otherks | 110082 | Bacillaene biosynthetic gene cluster (100% of genes show similarity) | 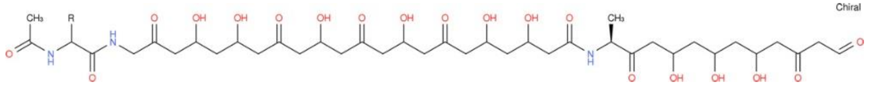 |
| Cluster 8 | Transatpks-Nrps | 80362 | Fengycin biosynthetic gene cluster (86% of genes show similarity) | 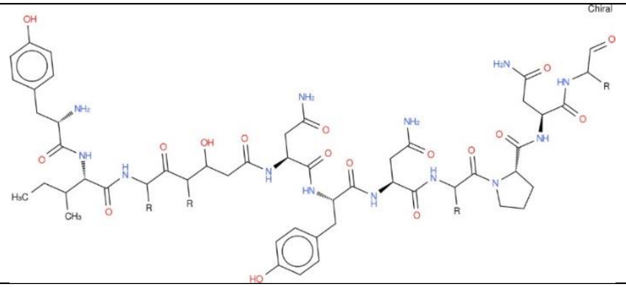 |
| Cluster 9 | Other | 41418 | Bacilysin biosynthetic gene cluster (100% of genes show similarity) | - |

Supplementary Table S3. Continued.

| Strain clusters | Type | Length (bp) | Most similar known clusters | Predicted core clusters |
|---|---|---|---|---|
| **III-1** | | | | |
| Cluster 10 | Sactipeptide-Head to tail | 21612 | Subtilosin A biosynthetic gene cluster (100% of genes show similarity) | - |
| Cluster 11 | Nrps | 49738 | Bacillibactin biosynthetic gene cluster (100% of genes show similarity) | 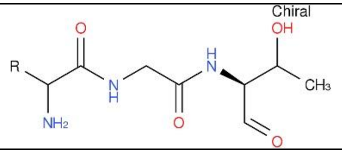 |
| Cluster 12 | T3pks | 41097 | - | - |
| Cluster 13 | Terpene | 21898 | - | - |
| Cluster 14 | Nrps | 23006 | Plipastatin biosynthetic gene cluster (38% of genes show similarity) | - |
| Cluster 15 | Nrps | 65394 | Surfactin biosynthetic gene cluster (86% of genes show similarity) | 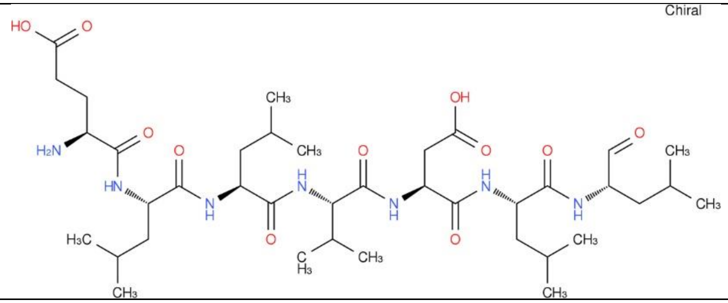 |
| Cluster 16 | Transatpks | 48051 | Phormidolide biosynthetic gene cluster (17% of genes show similarity) | 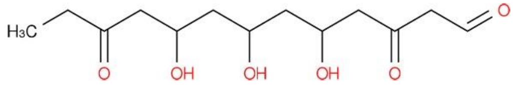 |
| Cluster 17 | Transatpks-T1pks-Otherks | 32386 | Phormidolide biosynthetic gene cluster (21% of genes show similarity) | 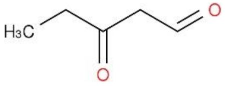 |

Supplementary Table S3. Continued.

| Strain clusters | Type | Length (bp) | Most similar known clusters | Predicted core clusters |
|---|---|---|---|---|
| **NRRL B-41618** | | | | |
| Cluster 1 | Transatpks-Nrps | 90713 | Fengycin biosynthetic gene cluster (80% of genes show similarity) | 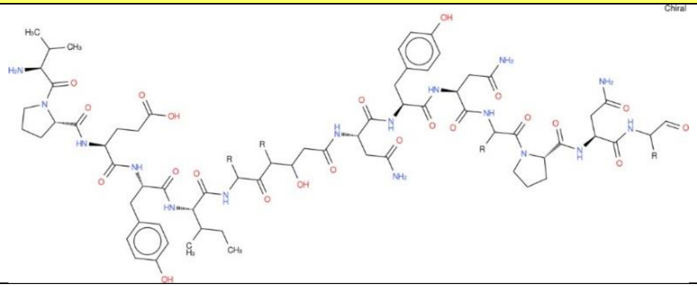 |
| Cluster 2 | Transatpks | 5608 | - | - |
| Cluster 3 | Nrps | 14364 | Plipastatin biosynthetic gene cluster (30% of genes show similarity) | 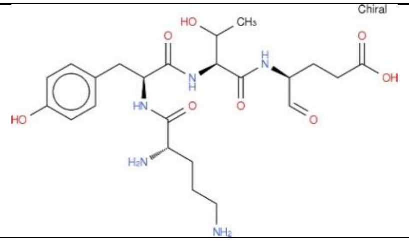 |
| Cluster 4 | Nrps | 65395 | Surfactin biosynthetic gene cluster (86% of genes show similarity) | 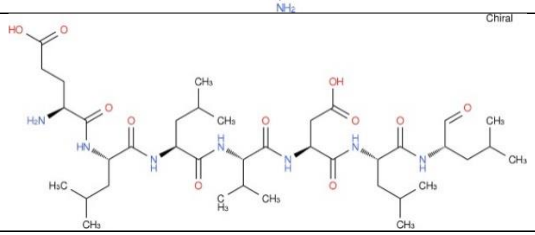 |
| Cluster 5 | Transatpks | 8190 | Elansolid biosynthetic gene cluster (10% of genes show similarity) | 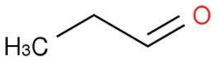 |
| Cluster 6 | Nrps | 1122 | - | - |
| Cluster 7 | Other | 41418 | Bacilysin biosynthetic gene cluster (100% of genes show similarity) | - |
| Cluster 8 | Sactipeptide-Head to tail | 21612 | Subtilosin A biosynthetic gene cluster (100% of genes show similarity) | - |

Supplementary Table S3. Continued.

| Strain clusters | Type | Length (bp) | Most similar known clusters | Predicted core clusters |
|---|---|---|---|---|
| **NRRL B-41618** | | | | |
| Cluster 9 | Nrps | 49738 | Bacillibactin biosynthetic gene cluster (100% of genes show similarity) | 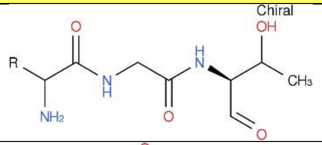 |
| Cluster 10 | Transatpks-Otherks-Nrps | 110097 | Bacillaene biosynthetic gene cluster (100% of genes show similarity) | 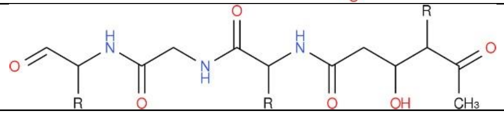 |
| Cluster 11 | Terpene | 20806 | - | - |
| Cluster 12 | Nrps | 22596 | Plipastatin biosynthetic gene cluster (38% of genes show similarity) | - |
| Cluster 13 | Terpene | 21898 | - | - |
| Cluster 14 | Transatpks-T1pks-Otherks | 32280 | Thiomarinol biosynthetic gene cluster (8% of genes show similarity) | 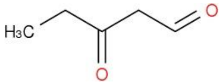 |
| Cluster 15 | T3pks | 41097 | - | - |
| Cluster 16 | Transatpks | 46509 | Myxovirescin biosynthetic gene cluster (13% of genes show similarity) | 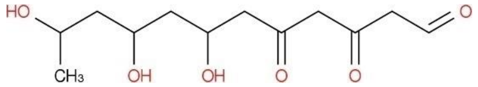 |

Supplementary Table S3. Continued.

| Strain clusters | Type | Length (bp) | Most similar known clusters | Predicted core clusters |
|---|---|---|---|---|
| **NRRL B-41617** | | | | |
| Cluster 1 | T3pks | 41097 | - | - |
| Cluster 2 | Terpene | 21898 | - | - |
| Cluster 3 | Nrps | 22905 | Plipastatin biosynthetic gene cluster (38% of genes show similarity) | - |
| Cluster 4 | Transatpks-T1pks-Otherks | 32259 | Phormidolide biosynthetic gene cluster (21% of genes show similarity) | 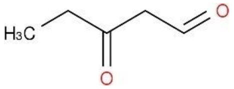 |
| Cluster 5 | Nrps | 10181 | Plipastatin biosynthetic gene cluster (23% of genes show similarity) | 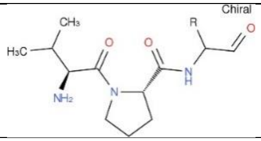 |
| Cluster 6 | Transatpks-Nrps | 63211 | Fengycin biosynthetic gene cluster (80% of genes show similarity) | 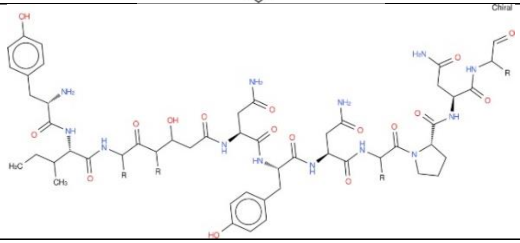 |
| Cluster 7 | Terpene | 20806 | - | - |
| Cluster 8 | Nrps-Transatpks-Otherks | 110093 | Bacillaene biosynthetic gene cluster (100% of genes show similarity) | 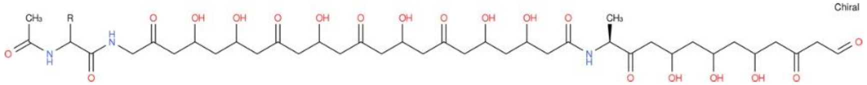 |
| Cluster 9 | Transatpks | 8405 | Elansolid biosynthetic gene cluster (10% of genes show similarity) | 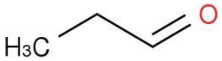 |
| Cluster 10 | Transatpks | 2083 | - | - |
| Cluster 11 | Nrps | 65394 | Surfactin biosynthetic gene cluster (86% of genes show similarity) | 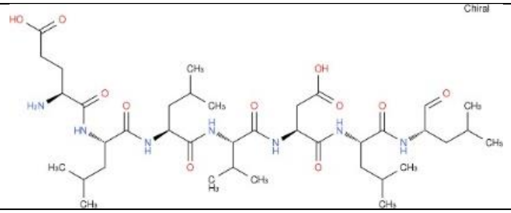 |

Supplementary Table S3. Continued.

| Strain clusters | Type | Length (bp) | Most similar known clusters | Predicted core clusters |
|---|---|---|---|---|
| **NRRL B-41617** | | | | |
| Cluster 12 | Other | 41418 | Bacilysin biosynthetic gene cluster (100% of genes show similarity) | - |
| Cluster 13 | Transatpks | 46517 | Myxovirescin biosynthetic gene cluster (13% of genes show similarity) | 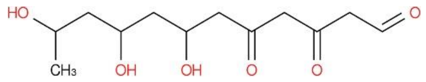 |
| Cluster 14 | Nrps | 49738 | Bacillibactin biosynthetic gene cluster (100% of genes show similarity) | 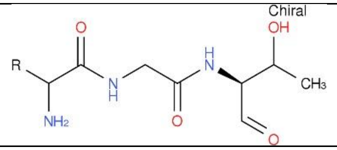 |
| Cluster 15 | Sactipeptide-Head to tail | 21612 | Subtilosin A biosynthetic gene cluster (100% of genes show similarity) | - |
| Cluster 16 | Nrps | 15038 | Plipastatin biosynthetic gene cluster (30% of genes show similarity) | 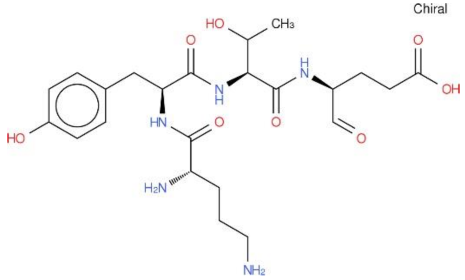 |

Supplementary Table S3. Continued.

| Strain clusters | Type | Length (bp) | Most similar known clusters | Predicted core clusters |
|---|---|---|---|---|
| **BFOA2 (72)** | | | | |
| Cluster 1 | Nrps-Transatpks-Otherks | 110091 | Bacillaene biosynthetic gene cluster (100% of genes show similarity) | 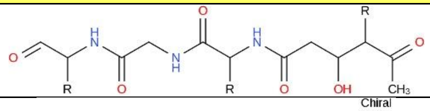 |
| Cluster 2 | Nrps | 33415 | Fengycin biosynthetic gene cluster (80% of genes show similarity) | 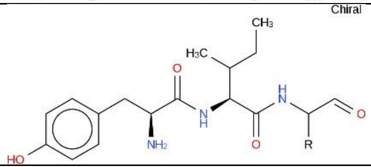 |
| Cluster 3 | Sactipeptide-Head_to_tail | 21612 | Subtilosin A biosynthetic gene cluster (100% of genes show similarity) | - |
| Cluster 4 | Other | 41418 | Bacilysin biosynthetic gene cluster (100% of genes show similarity) | - |
| Cluster 5 | Bacteriocin | 12183 | - | - |
| Cluster 6 | T3pks | 41097 | - | - |
| Cluster 7 | Terpene | 21898 | - | - |
| Cluster 8 | Nrps | 65395 | Surfactin biosynthetic gene cluster (86% of genes show similarity) | 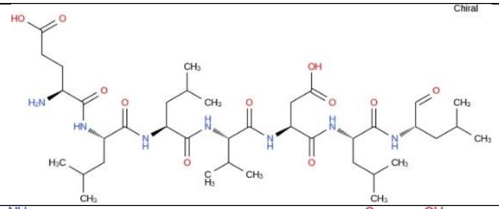 |
| Cluster 9 | Transatpks-Nrps | 61278 | Kijanimicin biosynthetic gene cluster (4% of genes show similarity) | 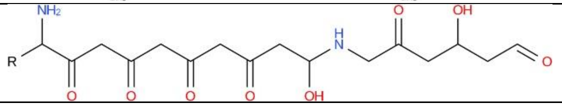 |
| Cluster 10 | Terpene | 20806 | - | - |
| Cluster 11 | Nrps | 43656 | Bacillibactin biosynthetic gene cluster (100% of genes show similarity) | 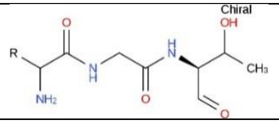 |
| Cluster 12 | Nrps | 23268 | Plipastatin biosynthetic gene cluster (46% of genes show similarity) | - |

Supplementary Table S3. Continued.

| Strain clusters | Type | Length (bp) | Most similar known clusters | Predicted core clusters |
|---|---|---|---|---|
| **BFOA2 (72)** | | | | |
| Cluster 13 | Nrps | 13200 | Plipastatin biosynthetic gene cluster (38% of genes show similarity) | 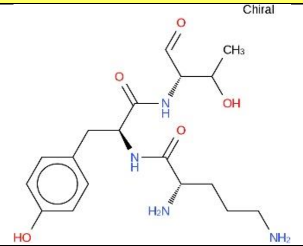 |
| Cluster 14 | Nrps | 10296 | Plipastatin biosynthetic gene cluster (15% of genes show similarity) | 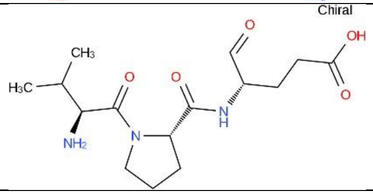 |

Supplementary Table S3. Continued.

| Strain clusters | Type | Length (bp) | Most similar known clusters | Predicted core clusters |
|---|---|---|---|---|
| **BFOA1 (86)** | | | | |
| Cluster 1 | Terpene | 20806 | - | - |
| Cluster 2 | Nrps-Transatpks-Otherks | 110091 | Bacillaene biosynthetic gene cluster (100% of genes show similarity) | 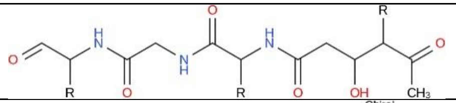 |
| Cluster 3 | Nrps | 33415 | Fengycin biosynthetic gene cluster (80% of genes show similarity) | 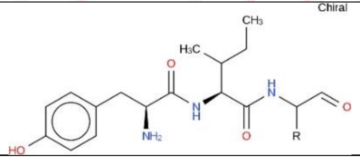 |
| Cluster 4 | Sactipeptide-Head to tail | 21612 | Subtilosin A biosynthetic gene cluster (100% of genes show similarity) | - |
| Cluster 5 | Other | 41418 | Bacilysin biosynthetic gene cluster (100% of genes show similarity) | - |
| Cluster 6 | Bacteriocin | 12183 | - | - |
| Cluster 7 | Transatpks-Nrps | 61278 | Kijanimicin biosynthetic gene cluster (4% of genes show similarity) | 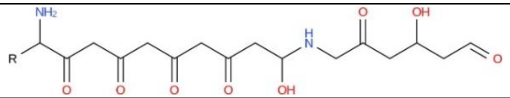 |
| Cluster 8 | T3pks | 41097 | - | - |
| Cluster 9 | Terpene | 21898 | - | - |
| Cluster 10 | Nrps | 25324 | Surfactin biosynthetic gene cluster (43% of genes show similarity) | - |
| Cluster 11 | Nrps | 28547 | Surfactin biosynthetic gene cluster (47% of genes show similarity) | 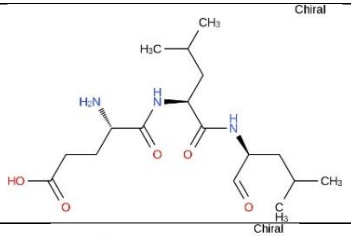 |
| Cluster 12 | Nrps | 43656 | Bacillibactin biosynthetic gene cluster (100% of genes show similarity) | 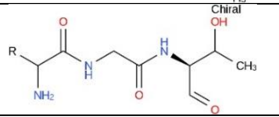 |

Supplementary Table S3. Continued.

| Strain clusters | Type | Length (bp) | Most similar known clusters | Predicted core clusters |
|---|---|---|---|---|
| **BFOA1 (86)** | | | | |
| Cluster 13 | Nrps | 23268 | Plipastatin biosynthetic gene cluster (46% of genes show similarity) | - |
| Cluster 14 | Nrps | 13615 | Fengycin biosynthetic gene cluster (26% of genes show similarity) | 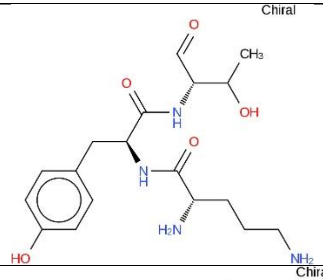  |
| Cluster 15 | Nrps | 10296 | Plipastatin biosynthetic gene cluster (15% of genes show similarity) | 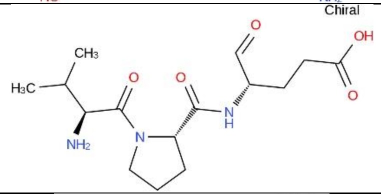  |
| Cluster 16 | Nrps | 9326 | Surfactin biosynthetic gene cluster (8% of genes show similarity) | 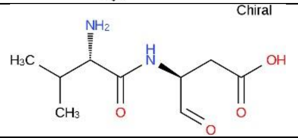  |
| Cluster 17 | Other | 1009 | - | - |

Supplementary Table S3. Continued.

| Strain clusters | Type | Length (bp) | Most similar known clusters | Predicted core clusters |
|---|---|---|---|---|
| **LNXM37** | | | | |
| Cluster 1 | Other | 41418 | Bacilysin biosynthetic gene cluster (100% of genes show similarity) | - |
| Cluster 2 | Sactipeptide-Head to tail | 21612 | Subtilosin A biosynthetic gene cluster (100% of genes show similarity) | - |
| Cluster 3 | Nrps | 49738 | Bacillibactin biosynthetic gene cluster (100% of genes show similarity) | 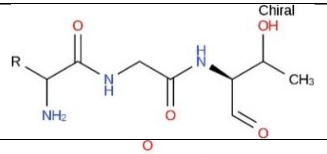 |
| Cluster 4 | Nrps-Transatpks-Otherks | 110098 | Bacillaene biosynthetic gene cluster (100% of genes show similarity) | 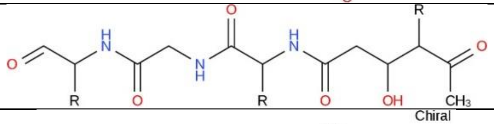 |
| Cluster 5 | Nrps | 33354 | Fengycin biosynthetic gene cluster (80% of genes show similarity) | 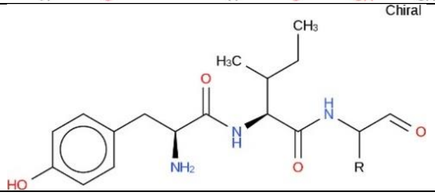 |
| Cluster 6 | T3pks | 41097 | - | - |
| Cluster 7 | Terpene | 21898 | - | - |
| Cluster 8 | Nrps | 26072 | Surfactin biosynthetic gene cluster (43% of genes show similarity) | - |
| Cluster 9 | Terpene | 20806 | - | - |
| Cluster 10 | Lantipeptide | 22597 | - | - |
| Cluster 11 | Nrps | 21754 | Plipastatin biosynthetic gene cluster (30% of genes show similarity) | - |
| Cluster 12 | Nrps | 28299 | Surfactin biosynthetic gene cluster (47% of genes show similarity) | 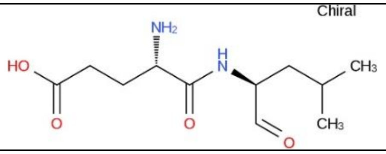 |

Supplementary Table S3. Continued.

| Strain clusters | Type | Length (bp) | Most similar known clusters | Predicted core clusters |
|---|---|---|---|---|
| **LNXM37** | | | | |
| Cluster 13 | Nrps | 13179 | Plipastatin biosynthetic gene cluster (23% of genes show similarity) | 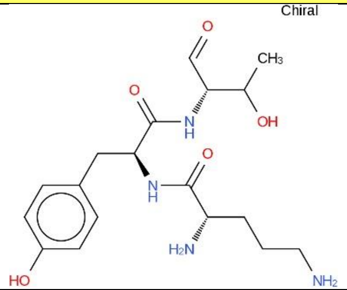 |
| Cluster 14 | Nrps | 9879 | Plipastatin biosynthetic gene cluster (15% of genes show similarity) | 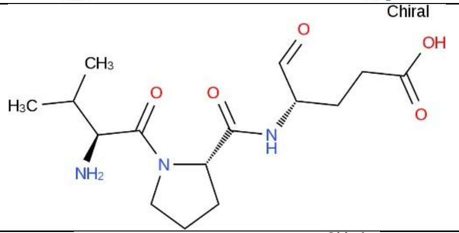 |
| Cluster 15 | Nrps | 9291 | Surfactin biosynthetic gene cluster (8% of genes show similarity) | 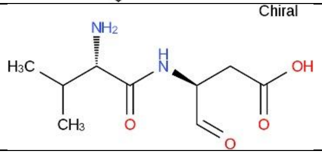 |

Supplementary Table S3. Continued.

| Strain clusters | Type | Length (bp) | Most similar known clusters | Predicted core clusters |
|---|---|---|---|---|
| **BFOA3 (36)** | | | | |
| Cluster 1 | Nrps-Transatpks-Otherks | 110091 | Bacillaene biosynthetic gene cluster (100% of genes show similarity) | 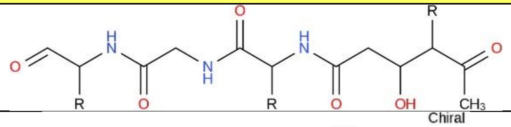 |
| Cluster 2 | Nrps | 33415 | Fengycin biosynthetic gene cluster (80% of genes show similarity) | 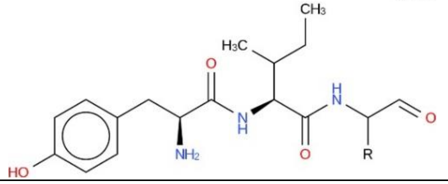 |
| Cluster 3 | Sactipeptide-Head to tail | 21612 | Subtilosin A biosynthetic gene cluster (100% of genes show similarity) | - |
| Cluster 4 | Other | 41418 | Bacilysin biosynthetic gene cluster (100% of genes show similarity) | - |
| Cluster 5 | Bacteriocin | 12183 | - | - |
| Cluster 6 | Transatpks-Nrps | 61278 | Kijanimicin biosynthetic gene cluster (4% of genes show similarity) | 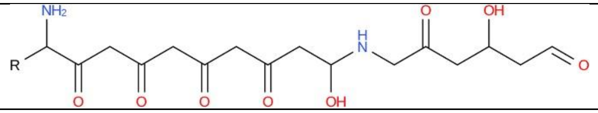 |
| Cluster 7 | T3pks | 41097 | - | - |
| Cluster 8 | Terpene | 21898 | - | - |
| Cluster 9 | Terpene | 20806 | - | - |
| Cluster 10 | Nrps | 25324 | Surfactin biosynthetic gene cluster (43% of genes show similarity) | - |
| Cluster 11 | Nrps | 28547 | Surfactin biosynthetic gene cluster (47% of genes show similarity) | 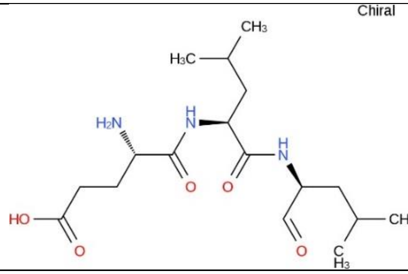 |

Supplementary Table S3. Continued.

| Strain clusters | Type | Length (bp) | Most similar known clusters | Predicted core clusters |
|---|---|---|---|---|
| **BFOA3 (36)** | | | | |
| Cluster 12 | Nrps | 43656 | Bacillibactin biosynthetic gene cluster (100% of genes show similarity) | 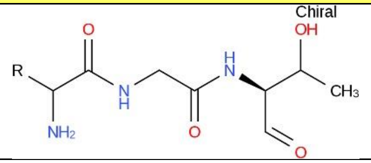 |
| Cluster 13 | Nrps | 23268 | Plipastatin biosynthetic gene cluster (46% of genes show similarity) | - |
| Cluster 14 | Nrps | 13200 | Plipastatin biosynthetic gene cluster (38% of genes show similarity) | 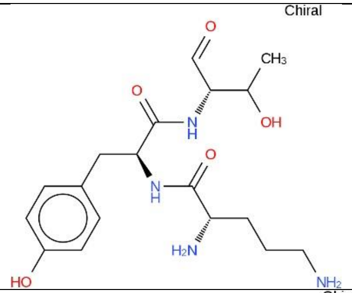 |
| Cluster 15 | Nrps | 10296 | Plipastatin biosynthetic gene cluster (15% of genes show similarity) | 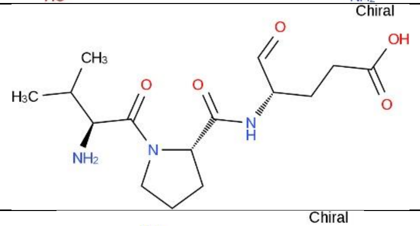 |
| Cluster 16 | Nrps | 9326 | Surfactin biosynthetic gene cluster (8% of genes show similarity) | 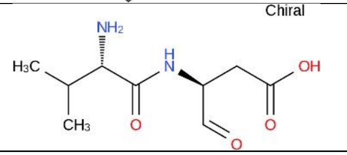 |
| Cluster 17 | Other | 1009 | - | - |

Supplementary Table S3. Continued.

| Strain clusters | Type | Length (bp) | Most similar known clusters | Predicted core clusters |
|---|---|---|---|---|
| **LNXM78** | | | | |
| Cluster 1 | Nrps | 49738 | Bacillibactin biosynthetic gene cluster (100% of genes show similarity) | 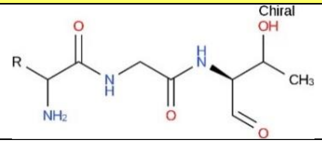 |
| Cluster 2 | Sactipeptide-Head to tail | 21612 | Subtilosin A biosynthetic gene cluster (100% of genes show similarity) | - |
| Cluster 3 | Other | 41418 | Bacilysin biosynthetic gene cluster (100% of genes show similarity) | - |
| Cluster 4 | T3pks | 41097 | - | - |
| Cluster 5 | Terpene | 20806 | - | - |
| Cluster 6 | Nrps-Transatpks-Otherks | 110098 | Bacillaene biosynthetic gene cluster (100% of genes show similarity) | 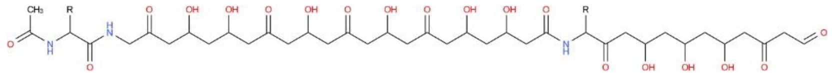 |
| Cluster 7 | Nrps | 65395 | Surfactin biosynthetic gene cluster (86% of genes show similarity) | 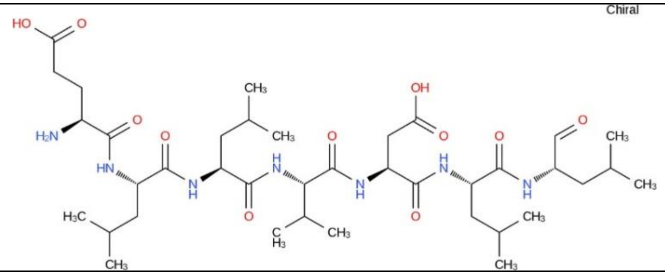 |
| Cluster 8 | Terpene | 21898 | - | - |
| Cluster 9 | Lantipeptide | 21755 | - | - |
| Cluster 10 | Nrps | 33542 | Fengycin biosynthetic gene cluster (80% of genes show similarity) | 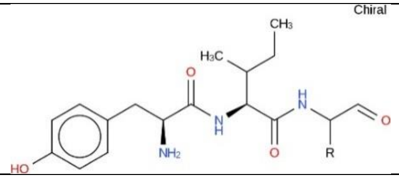 |
| Cluster 11 | Nrps | 21938 | Plipastatin biosynthetic gene cluster (30% of genes show similarity) | - |

Supplementary Table S3. Continued.

| Strain clusters | Type | Length (bp) | Most similar known clusters | Predicted core clusters |
|---|---|---|---|---|
| **LNXM78** | | | | |
| Cluster 12 | Nrps | 13531 | Plipastatin biosynthetic gene cluster (23% of genes show similarity) | 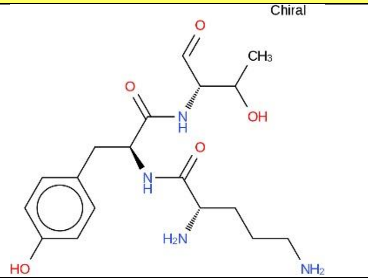 |
| Cluster 13 | Nrps | 10317 | Plipastatin biosynthetic gene cluster (23% of genes show similarity) | 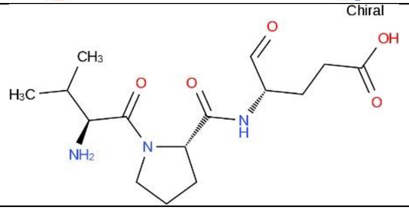 |
| Cluster 14 | Other | 1052 | - | - |
| Cluster 15 | Other | 1052 | - | - |

Supplementary Table S3. Continued.

| Strain clusters | Type | Length (bp) | Most similar known clusters | Predicted core clusters |
|---|---|---|---|---|
| **BFOA4 (B19)** | | | | |
| Cluster 1 | Terpene | 20806 | - | - |
| Cluster 2 | Other | 41418 | Bacilysin biosynthetic gene cluster (100% of genes show similarity) | - |
| Cluster 3 | Sactipeptide-Head to tail | 21612 | Subtilosin A biosynthetic gene cluster (100% of genes show similarity) | - |
| Cluster 4 | Transatpks-Otherks-Nrps | 105327 | Bacillaene biosynthetic gene cluster (100% of genes show similarity) | 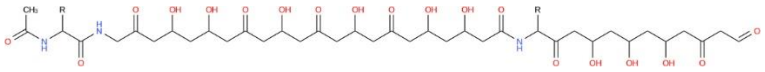 |
| Cluster 5 | Lantipeptide | 22597 | - | - |
| Cluster 6 | Nrps | 65395 | Surfactin biosynthetic gene cluster (86% of genes show similarity) | 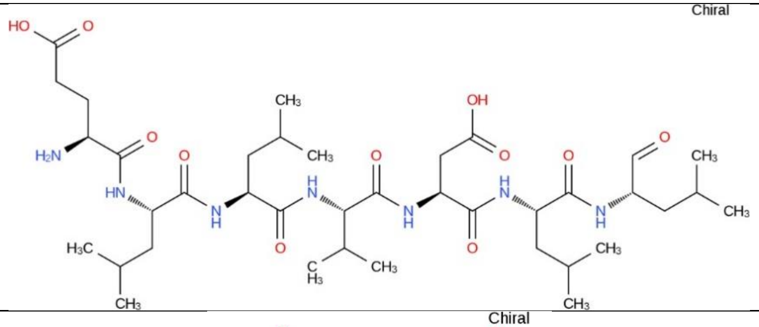 |
| Cluster 7 | Nrps | 49738 | Bacillibactin biosynthetic gene cluster (100% of genes show similarity) | 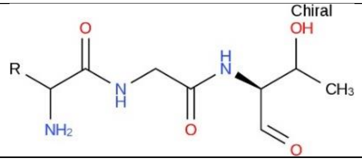 |
| Cluster 8 | T3pks | 41097 | - | - |
| Cluster 9 | Nrps | 33485 | Fengycin biosynthetic gene cluster (80% of genes show similarity) | 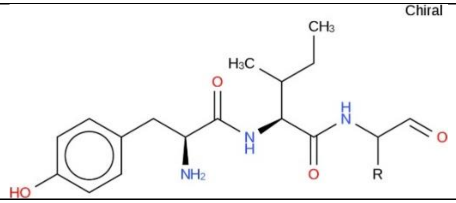 |
| Cluster 10 | Terpene | 21898 | - | - |

Supplementary Table S3. Continued.

| Strain clusters | Type | Length (bp) | Most similar known clusters | Predicted core clusters |
|---|---|---|---|---|
| **BFOA4 (B19)** | | | | |
| Cluster 11 | Nrps | 13174 | Plipastatin biosynthetic gene cluster (30% of genes show similarity) | 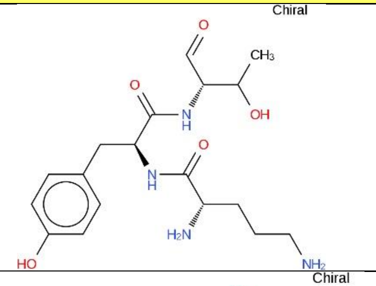 |
| Cluster 12 | Nrps | 9618 | Plipastatin biosynthetic gene cluster (23% of genes show similarity) | 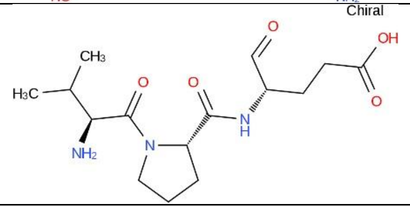 |
| Cluster 13 | Nrps | 5050 | Plipastatin biosynthetic gene cluster (23% of genes show similarity) | - |

Supplementary Table S3. Continued.

| Strain clusters | Type | Length (bp) | Most similar known clusters | Predicted core clusters |
|---|---|---|---|---|
| **RSCu-8D** | | | | |
| Cluster 1 | Nrps | 9233 | Plipastatin biosynthetic gene cluster (15% of genes show similarity) | 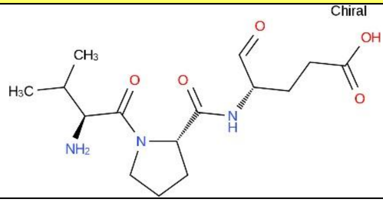 |
| Cluster 2 | Terpene | 20806 | - | - |
| Cluster 3 | Nrps-Transatpks-Otherks | 110097 | Bacillaene biosynthetic gene cluster (100% of genes show similarity) | 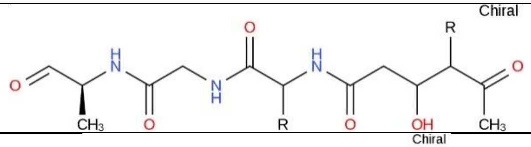 |
| Cluster 4 | Nrps | 57204 | Fengycin biosynthetic gene cluster (93% of genes show similarity) | 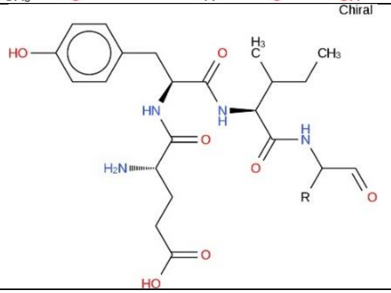 |
| Cluster 5 | Terpene | 21898 | - | - |
| Cluster 6 | T3pks | 41097 | - | - |
| Cluster 7 | Transatpks | 46339 | Myxovirescin biosynthetic gene cluster (13% of genes show similarity) | 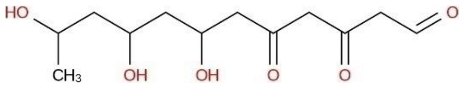 |
| Cluster 8 | Transatpks | 5388 | - | - |
| Cluster 9 | Nrps | 12848 | Plipastatin biosynthetic gene cluster (38% of genes show similarity) | 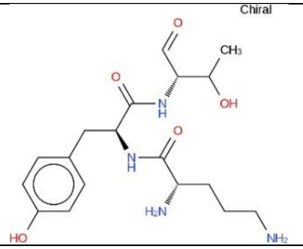 |

Supplementary Table S3. Continued.

| Strain clusters | Type | Length (bp) | Most similar known clusters | Predicted core clusters |
|---|---|---|---|---|
| **RSCu-8D** | | | | |
| Cluster 10 | Nrps | 49738 | Bacillibactin biosynthetic gene cluster (100% of genes show similarity) | 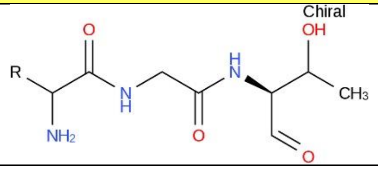 |
| Cluster 11 | Transatpks | 8471 | Elansolid biosynthetic gene cluster (10% of genes show similarity) | 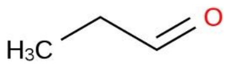 |
| Cluster 12 | Nrps | 65394 | Surfactin biosynthetic gene cluster (86% of genes show similarity) | 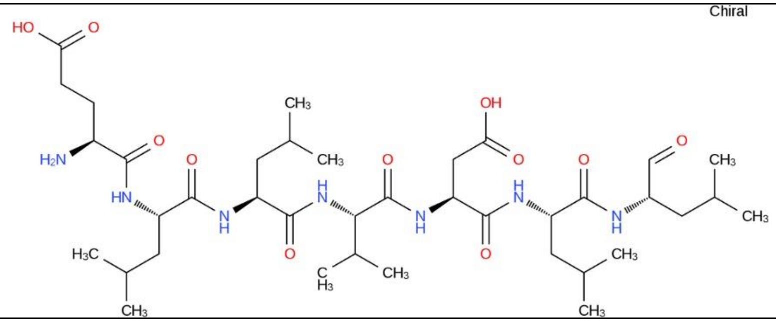 |
| Cluster 13 | Sactipeptide-Head to tail | 21612 | Subtilosin A biosynthetic gene cluster (100% of genes show similarity) | - |
| Cluster 14 | Other | 41418 | Bacilysin biosynthetic gene cluster (100% of genes show similarity) | - |
| Cluster 15 | Transatpks-T1pks-Otherks | 32875 | Thiomarinol biosynthetic gene cluster (8% of genes show similarity) | 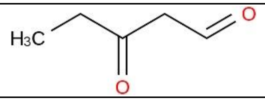 |

Supplementary Table S3. Continued.

| Strain clusters | Type | Length (bp) | Most similar known clusters | Predicted core clusters |
|---|---|---|---|---|
| **DGL6** | | | | |
| Cluster 1 | T3pks | 41097 | - | - |
| Cluster 2 | Terpene | 21898 | - | - |
| Cluster 3 | Nrps | 21680 | Plipastatin biosynthetic gene cluster (30% of genes show similarity) | - |
| Cluster 4 | Terpene | 20806 | - | - |
| Cluster 5 | Nrps-Transatpks-Otherks | 110103 | Bacillaene biosynthetic gene cluster (100% of genes show similarity) | 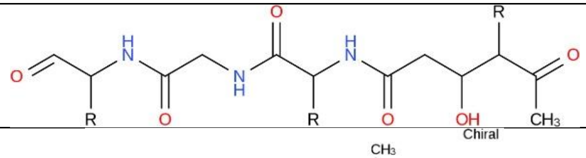 |
| Cluster 6 | Nrps | 33899 | Fengycin biosynthetic gene cluster (86% of genes show similarity) | 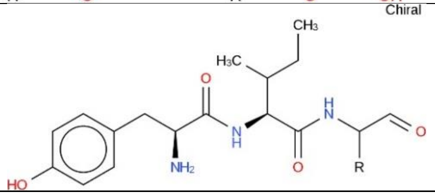 |
| Cluster 7 | Sactipeptide-Head to tail | 21612 | Subtilosin A biosynthetic gene cluster (100% of genes show similarity) | - |
| Cluster 8 | Other | 41418 | Bacilysin biosynthetic gene cluster (100% of genes show similarity) | - |
| Cluster 9 | Nrps | 49738 | Bacillibactin biosynthetic gene cluster (100% of genes show similarity) | 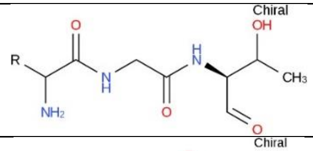 |
| Cluster 10 | Nrps | 14513 | Plipastatin biosynthetic gene cluster (30% of genes show similarity) | 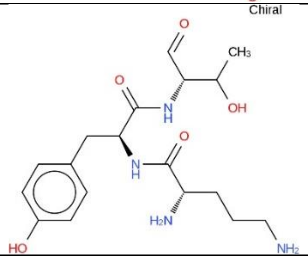 |

Supplementary Table S3. Continued.

| Strain clusters | Type | Length (bp) | Most similar known clusters | Predicted core clusters |
|---|---|---|---|---|
| **DGL6** | | | | |
| Cluster 11 | Nrps | 11005 | Plipastatin biosynthetic gene cluster (23% of genes show similarity) | 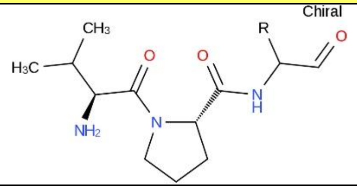 |
| Cluster 12 | Nrps | 1354 | - | - |

Supplementary Table S4. Compounds identified in *Bacillus halotolerans* isolates secreted fraction by LC-MS analysis.

| Rt (min) | HRESIMS | Molecular formula | Tentative identification | Structure | BFOA 4 | BFOA 3 | BFOA 2 | BFOA 1 | PGP activity | Biocontrol / antimicrobial activity | Herbicidal activity |
|---|---|---|---|---|---|---|---|---|---|---|---|
| 1.39 | 188.0699 | $C_{11}H_{11}NO_2$ | Indole propionic acid | 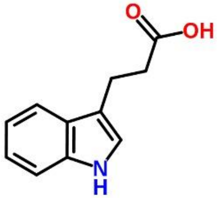 | + | + | + | + | Zhang and Davies (2016) | – | – |
| 1.71 | 197.1277 | $C_{10}H_{18}N_2O_2$ | Cyclo(L-Leu-N-methyl-L-Ala) | 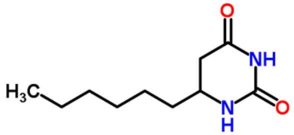 | – | + | + | + | – | – | – |
| 2.20 | 302.0819 | $C_{11}H_{17}N_3O_5S$ | 2-thio-5(N-methylaminomethyl)uridine | 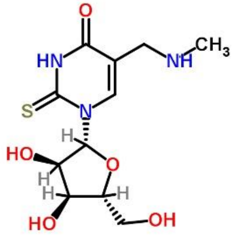 | – | – | + | + | – | – | – |
| 2.62 | 289.1550 | $C_{16}H_{22}N_2O_3$ | Inthomycin-A | 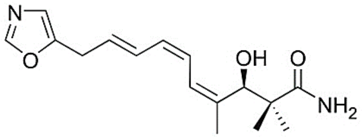 | + | + | + | + | – | Webb et al. (2008) | Webb et al. (2008) |
| 2.90 | 211.1433 | $C_{11}H_{20}N_2O_2$ | Cyclo(L-Val-L-Leu) | 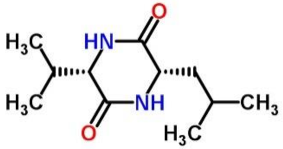 | + | + | + | + | – | – | – |
| 3.49 | 245.1270 | $C_{14}H_{18}N_2O_2$ | Cyclo(L-Val-L-Phe) | 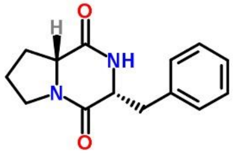 | – | + | – | + | – | Guo et al. (2011) | – |

Supplementary Table 4. Continued.

| Rt (min) | HRESIMS | Molecular formula | Tentative identification | Structure | BFOA 4 | BFOA 3 | BFOA 2 | BFOA 1 | PGP activity | Biocontrol / antimicrobial activity | Herbicidal activity |
|---|---|---|---|---|---|---|---|---|---|---|---|
| 4.46 | 340.2242 | $C_{17}H_{31}N_3O_4$ | Diprotin A (Isoleucyl Prolyl Isoleucine) | 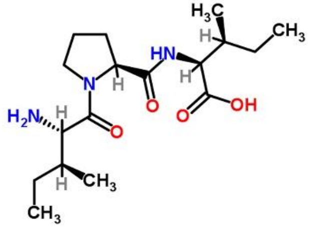 | + | + | + | + | – | – | – |
| 5.07 | 322.1295 | $C_{16}H_{21}NO_6$ | Methyl 3-acetamido-4,6-O-benzylidene-3-deoxy-β-D-glucopyranoside | 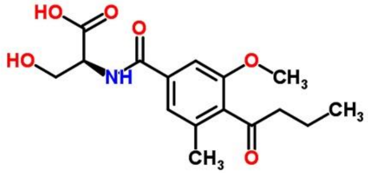 | + | – | – | – | – | – | – |
| 5.17 | 239.1187 | $C_{15}H_{16}N_2O$ | 3-(2-Butyl-5-oxazolyl)-1H-indole | 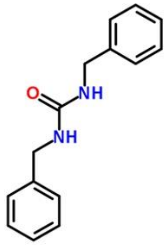 | + | – | – | – | – | – | – |
| 5.97 | 205.0966 | $C_{11}H_{14}N_2O_2$ | N-Acetylkynuramine | 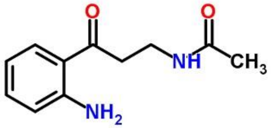 | + | – | – | – | – | – | – |
| 6.90 | 1139.6985 | $C_{60}H_{92}N_{12}O_{10}$ | Gramicidin S | 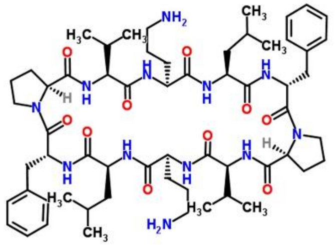 | + | + | + | + | – | Gause and Brazhnikova (1944) | – |

Supplementary Table 4. Continued.

| Rt (min) | HRESIMS | Molecular formula | Tentative identification | Structure | BFOA 4 | BFOA 3 | BFOA 2 | BFOA 1 | PGP activity | Biocontrol / antimicrobial activity | Herbicidal activity |
|---|---|---|---|---|---|---|---|---|---|---|---|
| 7.28 | 406.2300 | $C_{16}H_{33}N_5O_7$ | 5-Deoxybutirosamine | 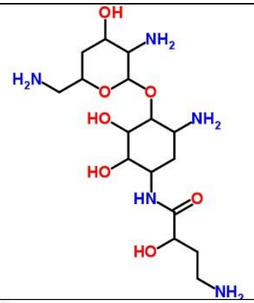 | + | – | – | – | – | Taylor and Schmitz (1976) | – |
| 7.33 | 1475.8043 | $C_{73}H_{112}N_{12}O_{20}$ | Plipastatin A2 | 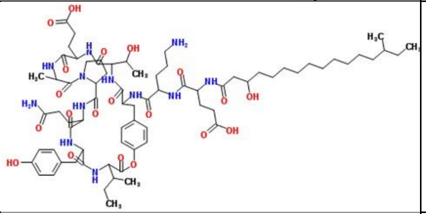 | + | + | + | + | – | Umezawa et al. (1986) | – |
| 7.44 | 1125.6832 | $C_{59}H_{90}N_{12}O_{10}$ | Gramicidin S2 | 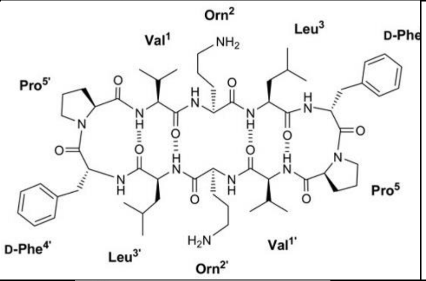 | + | + | + | + | – | Solanas et al. (2009) | – |
| 7.51 | 830.4935 | $C_{44}H_{65}N_9O_7$ | Gramicidin-J | 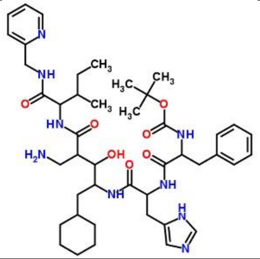 | + | + | + | + | – | Hunter and Schwartz (1967) | – |

Supplementary Table 4. Continued.

| Rt (min) | HRESIMS | Molecular formula | Tentative identification | Structure | BFOA 4 | BFOA 3 | BFOA 2 | BFOA 1 | PGP activity | Biocontrol / antimicrobial activity | Herbicidal activity |
|---|---|---|---|---|---|---|---|---|---|---|---|
| 7.61 | 1461.7884 | $C_{72}H_{110}N_{12}O_{20}$ | Plipastatin A1 | 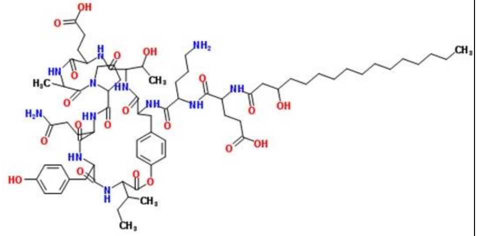 | + | – | + | + | – | Ma and Hu (2018) | – |
| 7.66 | 1489.8196 | $C_{74}H_{114}N_{12}O_{20}$ | Plipastatin B1 | 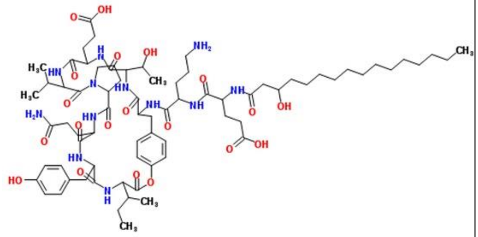 | + | – | + | + | – | Tsuge et al. (1996) | – |
| 7.97 | 845.4376 | $C_{35}H_{62}N_{10}O_{14}$ | Rotihibin-B | 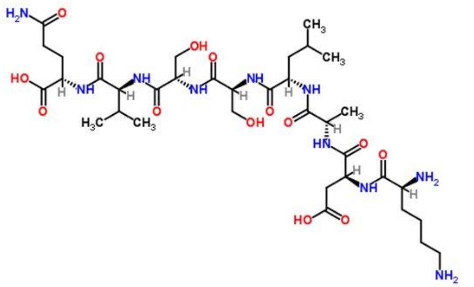 | + | – | – | – | Fukuchi et al. 1995 | – | – |
| 8.39 | 961.6161 | $C_{41}H_{82}N_{14}O_{12}$ | Galantin-I | 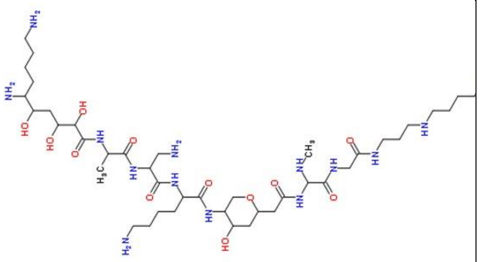 | + | – | – | – | – | Sakai and Ohfune (1992) | – |

Supplementary Table 4. Continued.

| Rt (min) | HRESIMS | Molecular formula | Tentative identification | Structure | BFOA 4 | BFOA 3 | BFOA 2 | BFOA 1 | PGP activity | Biocontrol / antimicrobial activity | Herbicidal activity |
|---|---|---|---|---|---|---|---|---|---|---|---|
| 8.46 | 993.6427 | $C_{42}H_{86}N_{14}O_{13}$ | Lysinegalantin I | – | + | + | + | + | – | – | – |
| 8.57 | 1007.6763 | $C_{51}H_{92}N_8O_{12}$ | Pseudofactin I | 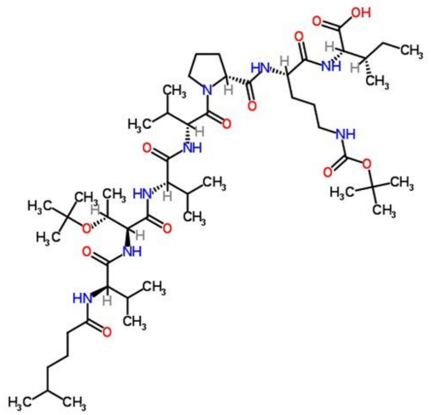 | + | + | + | + | – | Janek et al. (2012); Biniarz et al. (2015) | – |
| 8.62 | 1021.6916 | $C_{52}H_{94}N_8O_{12}$ | Pseudofactin II | - | | | | | | | |
| 9.28 | 339.3266 | $C_{22}H_{44}O_2$ | 20-methylheneicosanoic acid | 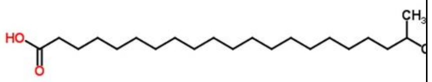 | – | + | + | + | – | – | – |

Supplementary Table S5. Compounds identified in *Bacillus halotolerans* strains secreted fraction by GC-MS analysis.

| Rt (min) | Kovat's Index | Molecular formula | Tentative identification | Structure | BFOA4 | BFOA3 | BFOA2 | BFOA1 | PGP activity | Biocontrol / antimicrobial activity | Herbicidal/ Insecticide activity |
|---|---|---|---|---|---|---|---|---|---|---|---|
| 4.69 | 873 | $C_5H_{11}N$ | Ethyl-2-methylpyrrolidine | 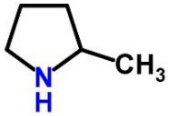 | + | + | – | – | – | – | – |
| 5.10 | 942 | $C_{10}H_{16}$ | Camphene | 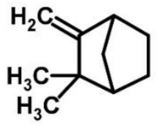 | + | + | – | – | – | – | – |
| 5.92 | 1003 | $C_{10}H_{22}$ | Decane | 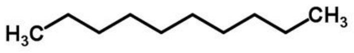 | – | + | – | – | – | – | – |
| 6.02 | 1011 | $C_{10}H_{14}$ | p-Cymene | 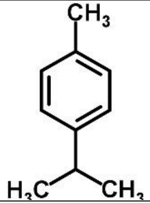 | – | + | – | – | – | – | – |
| 6.15 | 1014 | $C_{12}H_{24}$ | Trimethyl-1-nonene | 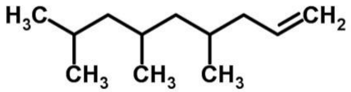 | + | + | – | – | – | – | – |
| 6.43 | 1018 | $C_{10}H_{16}$ | D-Limonene | 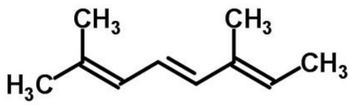 | – | – | + | + | – | – | Avenger Material Safety Data Sheet[1] |
| 6.78 | 1025 | $C_{10}H_{14}$ | o-Cymene | 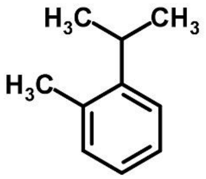 | + | + | – | – | – | – | – |

[1] http://nebula.wsimg.com/07de45c0af774ba73e06362ad1a56f06?AccessKeyId=C67FD801C8FC93742D64&disposition=0&alloworigin=1

Supplementary Table S5. Continued.

| Rt (min) | Kovat's Index | Molecular formula | Tentative identification | Structure | BFOA4 | BFOA3 | BFOA2 | BFOA1 | PGP activity | Biocontrol / antimicrobial activity | Herbicidal/ Insecticide activity |
|---|---|---|---|---|---|---|---|---|---|---|---|
| 6.87 | 1083 | $C_{10}H_{16}$ | α-terpinolene | 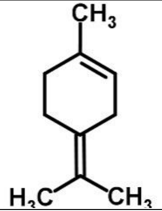 | + | + | + | – | – | – | – |
| 7.51 | 1109 | $C_{10}H_{16}O$ | cis-4-Caranone | 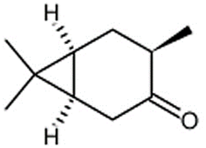 | + | + | – | – | – | – | – |
| 7.66 | 1115 | $C_{11}H_{24}$ | Undecane | 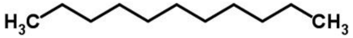 | + | + | – | – | – | – | – |
| 7.88 | 1151 | $C_{12}H_{26}$ | Dimethyldecane | 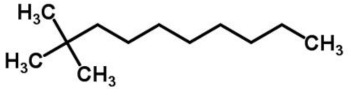 | + | + | – | – | – | – | – |
| 8.15 | 1158 | $C_{10}H_{18}O$ | Borneol | 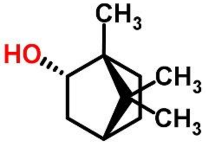 | + | + | – | – | – | – | Chemical Information[2] |
| 8.28 | 1170 | $C_{12}H_{26}$ | 2-methyl-undecane | 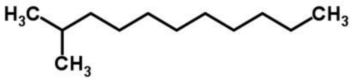 | + | + | – | – | – | – | – |
| 8.41 | 1179 | $C_{10}H_{16}O$ | Dihydrocarvone | 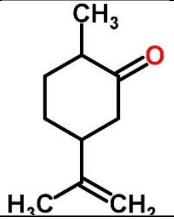 | + | + | – | – | – | – | – |

2 https://web.archive.org/web/20041107161247/http://sun.ars-grin.gov:8080/npgspub/xsql/duke/chemdisp.xsql?chemical=BORNEOL

Supplementary Table S5. Continued.

| Rt (min) | Kovat's Index | Molecular formula | Tentative identification | Structure | BFOA4 | BFOA3 | BFOA2 | BFOA1 | PGP activity | Biocontrol / antimicrobial activity | Herbicidal/ Insecticide activity |
|---|---|---|---|---|---|---|---|---|---|---|---|
| 8.57 | 1185 | $C_{10}H_{18}O$ | α-terpineol | 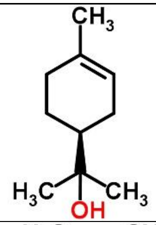 | + | + | – | – | – | – | – |
| 9.06 | 1201 | $C_{10}H_{16}O$ | trans-p-mentha-1(7),8-dien-2-ol | 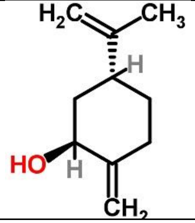 | + | + | – | – | – | – | – |
| 9.13 | 1209 | $C_{12}H_{26}$ | Dodecane | 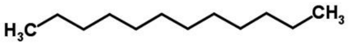 | + | + | – | – | – | – | – |
| 9.21 | 1216 | $C_{10}H_{16}O$ | Pulegone | 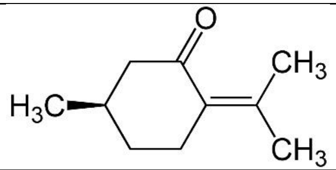 | + | + | – | – | – | – | Franzios et al. (1997) |
| 9.34 | 1234 | $C_{13}H_{28}$ | 2,3-dimethyl-undecane | 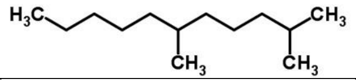 | + | + | + | – | – | – | – |
| 9.64 | 1271 | $C_{13}H_{24}$ | Tridecadiene | 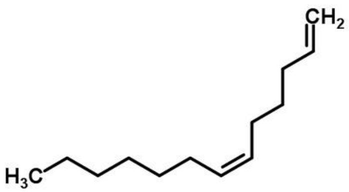 | + | + | + | – | – | – | – |
| 9.99 | 1298 | $C_{11}H_{22}O$ | 2-undecanone | 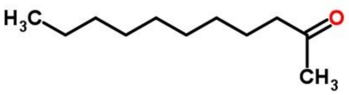 | + | + | – | – | – | – | Stephen et al. (2002) |

Supplementary Table S5. Continued.

| Rt (min) | Kovat's Index | Molecular formula | Tentative identification | Structure | BFOA4 | BFOA3 | BFOA2 | BFOA1 | PGP activity | Biocontrol / antimicrobial activity | Herbicidal/ Insecticide activity |
|---|---|---|---|---|---|---|---|---|---|---|---|
| 10.51 | 1313 | $C_{13}H_{28}$ | Tridecane | 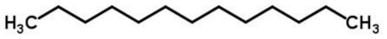 | + | + | + | – | – | – | – |
| 10.67 | 1319 | $C_{14}H_{30}$ | Tetramethyldecane | 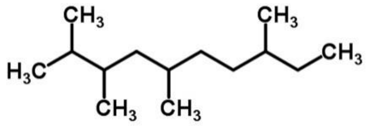 | + | + | + | – | – | – | – |
| 10.83 | 1349 | $C_{14}H_{30}$ | Methyltridecane | 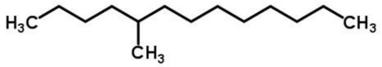 | + | + | + | – | – | – | – |
| 11.14 | 1381 | $C_{15}H_{32}$ | Farnesane | 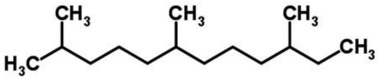 | – | + | + | – | – | – | – |
| 11.93 | 1411 | $C_{14}H_{26}$ | Tetradecadiene | 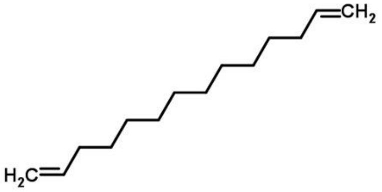 | + | + | + | – | – | – | – |
| 12.32 | 1417 | $C_{14}H_{30}$ | Tetradecane | 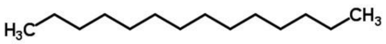 | – | + | + | – | – | – | – |
| 13.18 | 1423 | $C_{15}H_{24}$ | *trans*-Caryophyllene | 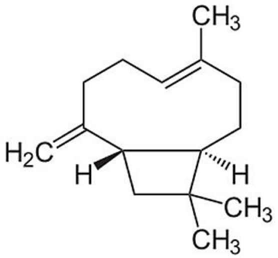 | – | + | + | – | – | – | – |
| 13.26 | 1439 | $C_{12}H_{24}$ | Cyclododecane | 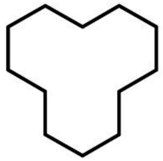 | – | – | + | – | – | – | – |

Supplementary Table S5. Continued.

| Rt (min) | Kovat's Index | Molecular formula | Tentative identification | Structure | BFOA4 | BFOA3 | BFOA2 | BFOA1 | PGP activity | Biocontrol / antimicrobial activity | Herbicidal/ Insecticide activity |
|---|---|---|---|---|---|---|---|---|---|---|---|
| 13.38 | 1477 | $C_{15}H_{24}$ | Germacrene D | 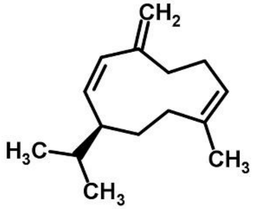 | – | – | + | – | – | Adio (2009) | Adio (2009) |
| 13.57 | 1523 | $C_{15}H_{26}O$ | 7-epi-cis-sesquisabinene | 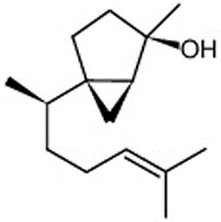 | – | – | + | – | – | – | – |
| 13.79 | 1563 | $C_{18}H_{36}O$ | Hexa-hydro-farnesyl | 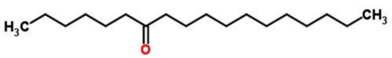 | – | + | + | – | – | – | – |
| 14.50 | 1573 | $C_{15}H_{26}O_2$ | Geranyl isovalerate | 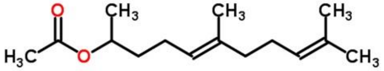 | – | + | – | – | – | – | – |
| 15.94 | 1557 | $C_{17}H_{36}$ | Trimethyltetradecane | 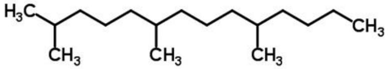 | – | – | + | – | – | – | – |
| 16.96 | 1612 | $C_{16}H_{34}$ | Hexadecane | 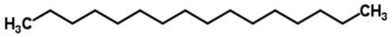 | – | – | + | – | – | – | – |
| 34.71 | 1939 | $C_{18}H_{18}$ | 1,5-Diphenyl-1,5-hexadiene | 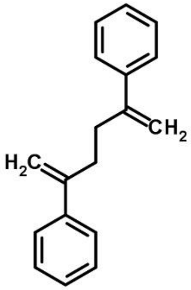 | – | – | + | + | – | – | – |

Supplementary Table S5. Continued.

| Rt (min) | Kovat's Index | Molecular formula | Tentative identification | Structure | BFOA4 | BFOA3 | BFOA2 | BFOA1 | PGP activity | Biocontrol / antimicrobial activity | Herbicidal/ Insecticide activity |
|---|---|---|---|---|---|---|---|---|---|---|---|
| 35.82 | 2244 | $C_{22}H_{42}O_4$ | Diisooctyl adipate | 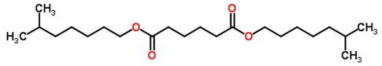 | – | – | + | + | – | – | – |
| 37.00 | 2704 | $C_{24}H_{38}O_4$ | Diisooctyl phthalate | 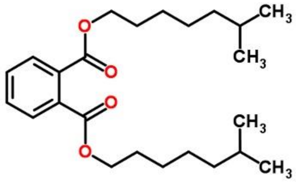 | – | – | + | – | – | – | – |

Supplementary Figure S1. Percentage of fungal (*Fusarium oxysporum* f. sp. *albedinis* strain LMA2) growth inhibition against *Bacillus halotolerans* isolates. Data presents mean ± standard error. Bars labelled with different letters are significantly different among the treatments at $p < 0.05$ using the Tukey's HSD test.

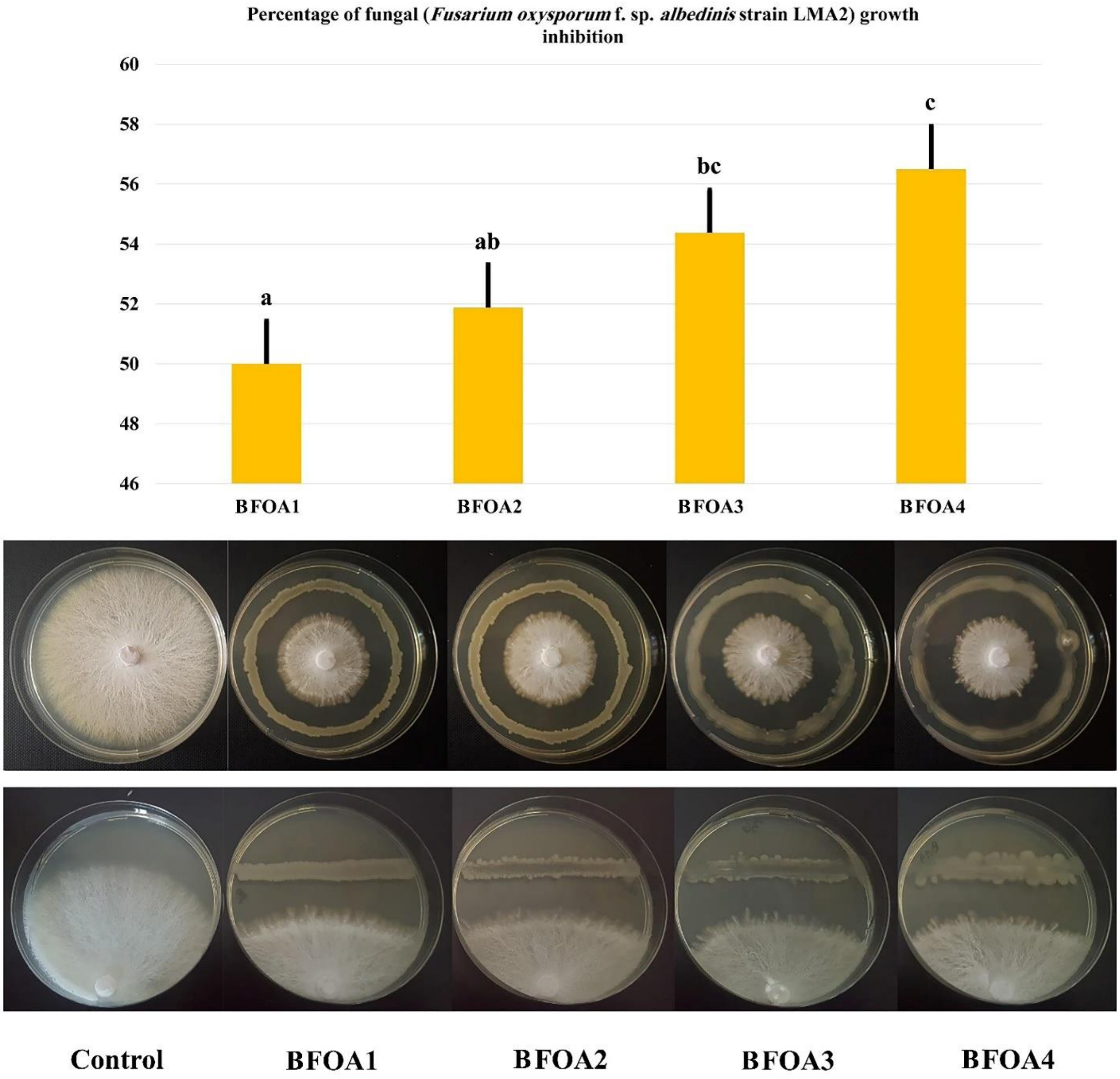

Supplementary Figure S2. *Fusarium oxysporum* f. sp. albedinis strains LMA3, LMA4 and LMA5 growth inhibition against *Bacillus halotolerans* isolates.

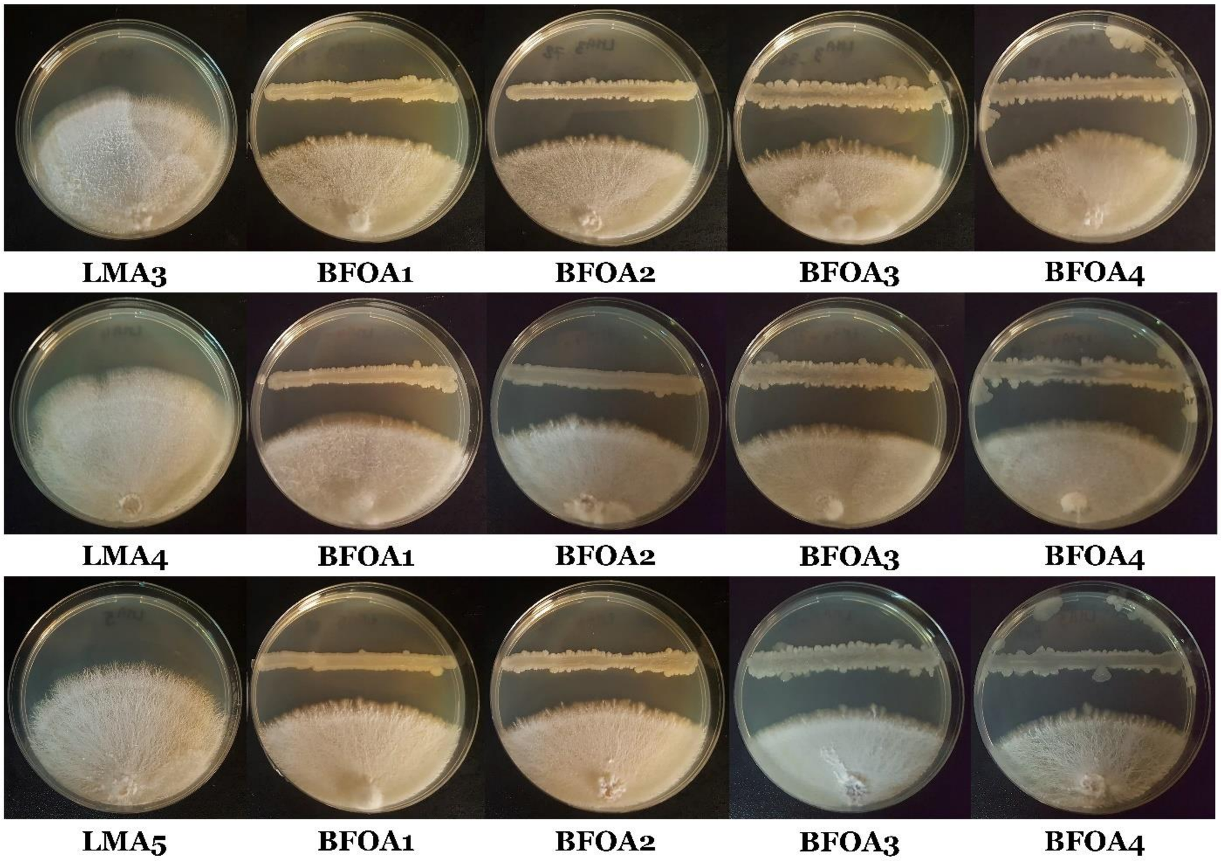

Supplementary Figure S3. Antagonistic effect of the biocontrol agent FOA4 against *Fusarium oxysporum* f. sp. radicis-lycopersici strain FORL. (A) Effects of FOA1-4 bacterial isolates on Fusarium oxysporum f. sp. radicis-lycopersici strain FORL tomato fruit rot severity as compared to tomato fruit controls. Data presents mean ± standard error. Bars labelled with different letters are significantly different among the treatments at $p < 0.05$ using the Tukey's HSD test. (B) Effect of preventive, concomitant, and curative biocontrol treatment on tomato fruits at 7 days post-inoculation with *Fusarium* oxysporum f. sp. radicis-lycopersici strain FORL.

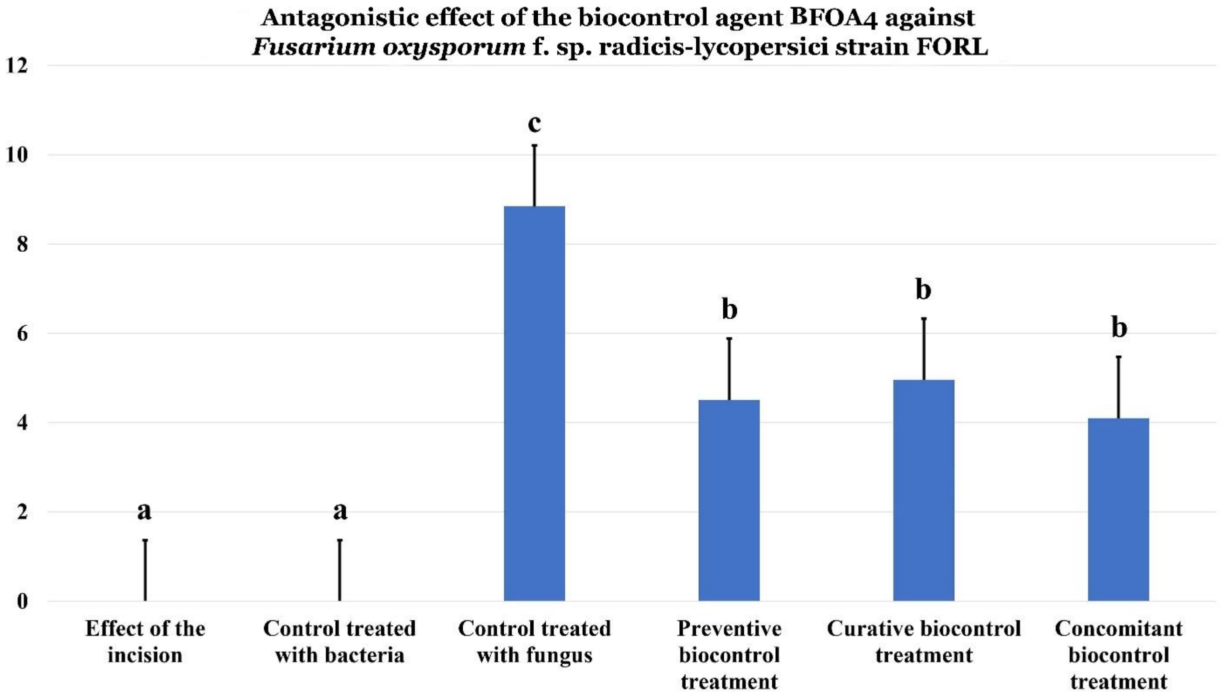


A

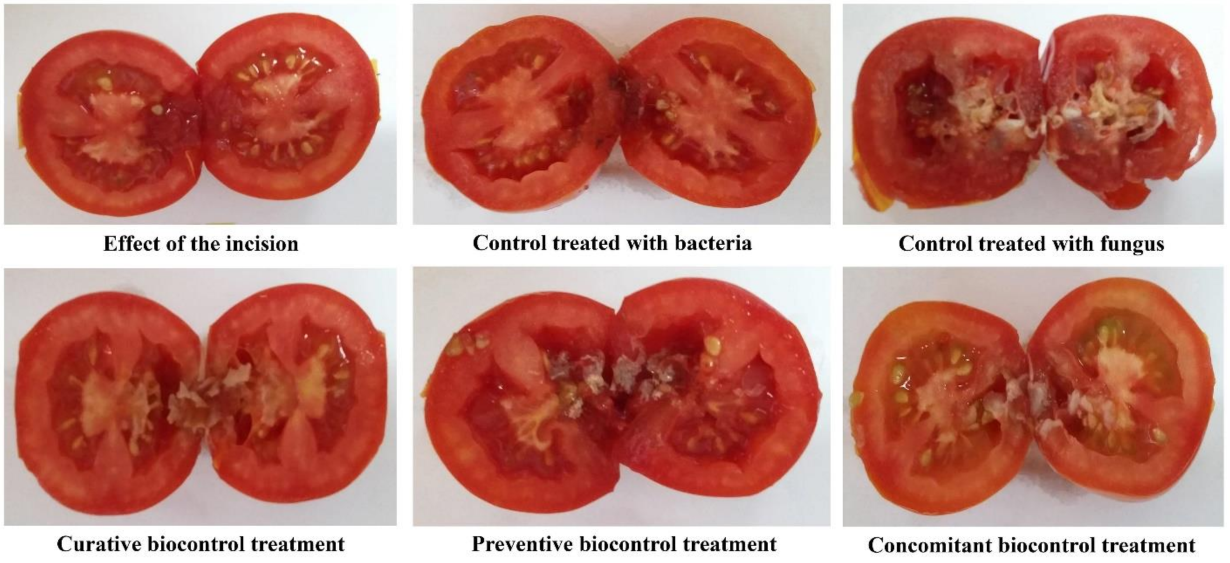


B

## References

Adio, A. M. (2009). Germacrenes A–E and related compounds: thermal, photochemical and acid induced transannular cyclizations. *Tetrahedron*. 65, 1533–1552 10.1016/j.tet.2008.11.050

Biniarz, P., Baranowska, G., Feder-Kubis, J. and Krasowska, A. (2015). The lipopeptides pseudofactin II and surfactin effectively decrease *Candida albicans* adhesion and hydrophobicity. *Antonie Leeuwenhoek*. 108, 343-353. doi: 10.1007/s10482-015-0486-3

Franzios, G., Mirotsou, M., Hatziapostolou, E., Kral, J., Scouras, Z. G., Mavragani-Tsipidou, P. (1997). Insecticidal and genotoxic activities of mint essential oils. *J. Agri. Food Chem*. 45 (7), 2690–2694. doi:10.1021/jf960685f

Fukuchi, N., Furihata, K., Nakayama, J., and Isogai, A. (1995). Rotihibins, novel plant growth regulators from *Streptomyces graminofaciens*. *J. Antibiot*. 48(9), 1004-1010. doi: 10.7164/antibiotics.48.1004

Gause, G. F., Brazhnikova, M. G. (1944). Gramicidin S and its use in the treatment of infected Wounds. Nature. 154(3918): 703–703, doi:10.1038/154703a0

Guo, X., Zheng, L., Zhou, W. et al. (2011). A case study on chemical defense based on quorum sensing: antibacterial activity of sponge-associated bacterium *Pseudoalteromonas* sp. NJ6-3-1 induced by quorum sensing mechanisms. *Ann Microbiol*. 61(2), 247–255. doi: 10.1007/s13213-010-0129-x

Hunter, F. E. and Schwartz, L. S. (1967). Tyrocidines and Gramicidin S (J1, J2). In: Gottlieb, D., Shaw, P. D. (eds) Antibiotics. Springer, Berlin, Heidelberg.

Janek, T., Lukaszewicz, M. and Krasowska, A. (2012). Antiadhesive activity of the biosurfactant pseudofactin II secreted by the arctic bacterium *Pseudomonas fluorescens* BD5. *BMC Microbiol*. 12:24.

Ma, Z. and Hu, J. (2018). Plipastatin A1 produced by a marine sediment-derived *Bacillus amyloliquefaciens* SH-B74 contributes to the control of gray mold disease in tomato. *3 Biotech*. 8(2):125. doi: 10.1007/s13205-018-1144-z

Sakai, N. and Ohfune, Y. (1992). Total synthesis of Galantin I. Acid-catalyzed cyclization of galantinic acid. *J. Am. Chem. Soc*. 114, 998-1010.

Solanas, C., de la Torre, B. G., Fernandez-Reyes, M., et al. (2009). Therapeutic Index of Gramicidin S is Strongly Modulated by D-Phenylalanine Analogues at the Beta-Turn. *J. Med. Chem*. 52, 664–674.

Stephen, J., Toth, Jr. and Buhler, W. G. (2002). North Carolina State University scientist discovers mosquito repellent in tomatoes. *Pesticide Broadcast*. 12: (5).

Taylor, H. D. and Schmitz, H. (1976). Antibiotics derived from a mutant of *Bacillus circulans*. *J Antibiot*. 29(5), 532-535.

Tsuge, K., Ano, T. and Shoda, M. (1996). Isolation of a gene essential for biosynthesis of the lipopeptide antibiotics plipastatin B1 and surfactin in *Bacillus subtilis* YB8. *Arch. Microbiol*. 165(4), 243-251.

Umezawa, H., Aoyagi, T., Nishikiori, T., Okuyama, A., Yamagishi, Y., Hamada, M. and Takeuchi, T. (1986). Plipastatins: new inhibitors of phospholipase A2, produced by *Bacillus cereus* BMG302-fF67. I. Taxonomy, production, isolation and preliminary characterization. *J Antibiot*. (Tokyo). 39 (6), 737-44.

Webb, M. R., Addie, M. S., Crawforth, C. M. et al. (2008). The syntheses of rac-inthomycin A, (+)-inthomycin B and (+)-inthomycin C using a unified synthetic approach. *Tetrahedron*. 64 (21), 4778-4791. doi: 10.1016/j.tet.2008.01.116

Zhang, L. S. and Davies, S. S. (2016). Microbial metabolism of dietary components to bioactive metabolites: opportunities for new therapeutic interventions. *Genome Med*. 8:46. doi: 10.1186/s13073-016-0296-x
